# Plasma Metabolomic Signatures of Chronic Obstructive Pulmonary Disease and the Impact of Genetic Variants on Phenotype-Driven Modules

**DOI:** 10.1089/nsm.2020.0009

**Published:** 2020-12-31

**Authors:** Lucas A. Gillenwater, Katherine A. Pratte, Brian D. Hobbs, Michael H. Cho, Yonghua Zhuang, Eitan Halper-Stromberg, Charmion Cruickshank-Quinn, Nichole Reisdorph, Irina Petrache, Wassim W. Labaki, Wanda K. O'Neal, Victor E. Ortega, Dean P. Jones, Karan Uppal, Sean Jacobson, Gregory Michelotti, Christine H. Wendt, Katerina J. Kechris, Russell P. Bowler

**Affiliations:** ^1^ National Jewish Health, Denver, Colorado, USA.; ^2^ Channing Division of Network Medicine, Brigham and Women's Hospital, Boston, Massachusetts, USA.; ^3^ Division of Pulmonary and Critical Care Medicine, Brigham and Women's Hospital, Boston, Massachusetts, USA.; ^4^ Department of Biostatistics and Informatics, Colorado School of Public Health, University of Colorado Anschutz Medical Campus, Aurora, Colorado, USA.; ^5^ Department of Pathology, Johns Hopkins University, Baltimore, Maryland, USA.; ^6^ Agilent Technologies, Santa Clara, California, USA.; ^7^ Skaggs School of Pharmacy and Pharmaceutical Sciences, University of Colorado Anschutz Medical Campus, Aurora, Colorado, USA.; ^8^ School of Medicine, University of Colorado, Aurora, Colorado, USA.; ^9^ Division of Pulmonary and Critical Care Medicine, University of Michigan, Ann Arbor, Michigan, USA.; ^10^ Lung Institute/Cystic Fibrosis Center, University of North Carolina at Chapel Hill, Chapel Hill, North Carolina, USA.; ^11^ Department of Internal Medicine, Center for Precision Medicine, Wake Forest School of Medicine, Winston-Salem, North Carolina, USA.; ^12^ Clinical Biomarkers Laboratory, Division of Pulmonary, Allergy, and Critical Care Medicine, Emory School of Medicine, Atlanta, Georgia, USA.; ^13^ Metabolon, Inc., Morrisville, North Carolina, USA.; ^14^ Department of Medicine, University of Minnesota and the VAMC, Minneapolis, Minnesota, USA.

**Keywords:** metabolomics, chronic obstructive pulmonary disease, metabolomic quantitative trait analysis, integrated omics, network analysis

## Abstract

**Background:** Small studies have recently suggested that there are specific plasma metabolic signatures in chronic obstructive pulmonary disease (COPD), but there have been no large comprehensive study of metabolomic signatures in COPD that also integrate genetic variants.

**Materials and Methods:** Fresh frozen plasma from 957 non-Hispanic white subjects in COPDGene was used to quantify 995 metabolites with Metabolon's global metabolomics platform. Metabolite associations with five COPD phenotypes (chronic bronchitis, exacerbation frequency, percent emphysema, post-bronchodilator forced expiratory volume at one second [FEV_1_]/forced vital capacity [FVC], and FEV_1_ percent predicted) were assessed. A metabolome-wide association study was performed to find genetic associations with metabolite levels. Significantly associated single-nucleotide polymorphisms were tested for replication with independent metabolomic platforms and independent cohorts. COPD phenotype-driven modules were identified in network analysis integrated with genetic associations to assess gene-metabolite-phenotype interactions.

**Results:** Of metabolites tested, 147 (14.8%) were significantly associated with at least 1 COPD phenotype. Associations with airflow obstruction were enriched for diacylglycerols and branched chain amino acids. Genetic associations were observed with 109 (11%) metabolites, 72 (66%) of which replicated in an independent cohort. For 20 metabolites, more than 20% of variance was explained by genetics. A sparse network of COPD phenotype-driven modules was identified, often containing metabolites missed in previous testing. Of the 26 COPD phenotype-driven modules, 6 contained metabolites with significant met-QTLs, although little module variance was explained by genetics.

**Conclusion:** A dysregulation of systemic metabolism was predominantly found in COPD phenotypes characterized by airflow obstruction, where we identified robust heritable effects on individual metabolite abundances. However, network analysis, which increased the statistical power to detect associations missed previously in classic regression analyses, revealed that the genetic influence on COPD phenotype-driven metabolomic modules was modest when compared with clinical and environmental factors.

## Introduction

Metabolites are low molecular weight (≤1500 Daltons) molecules, representing both endogenous and exogenous (environmentally derived) compounds, which play important roles in signaling, energy expenditure, reproduction, and growth. Metabolites vary greatly across individuals and can act as a unique identifier of an individual through time.^1^ Metabolite expression is thought to be the most proximal signature of health and disease, when compared to other omics (e.g., genomics, transcriptomics, and proteomics).^2^

Recently, there have been several reports suggesting the presence of characteristic metabolic signatures in the blood of individuals with lung diseases such as chronic obstructive pulmonary disease (COPD)^3–8^; however, these reports have typically included only a small number of subjects or a limited annotation of metabolic features (<500 metabolites). During the past few years, substantial gains have been made in metabolomics, using analytical chemistry techniques and advanced computational methods to characterize complex biological mixtures. The highly sensitive detection techniques of liquid chromatography tandem mass spectrometry (LC-MS/MS) quantify metabolites from a broad range of classes and are now automated to increase throughput, enabling large cohort-level epidemiological metabolome studies.^9,10^ These strategies have not yet been used on a large scale to study COPD.

COPD is characterized by progressive airflow limitation due to airway and/or alveolar abnormalities and is now the third most common cause of death worldwide,^11^ and is one of the leading causes of medical hospitalizations in the United States.^12^ Cigarette smoking is the greatest environmental risk factor, yet most smokers do not develop clinically important lung disease, and COPD heritability is estimated to be ∼37%.^13^ Furthermore, for those who do develop COPD, there are heterogeneous phenotypes, including emphysema, chronic bronchitis, and frequent COPD exacerbations (Supplementary Fig. S1).^14^ While clinical variables such as age, race, sex, and environmental factors like smoking and body mass index (BMI) have been useful in modeling disease severity, a large amount of unexplained variability in COPD severity remains.^15^ COPD is also associated with increased risk of non-pulmonary diseases independent of smoking history (e.g., cardiovascular disease, osteoporosis, depression, and cancer outside of the lung), suggesting the presence of systemic disturbances in metabolic pathways across comorbidities.^16^ The availability of large longitudinal cohorts for smokers with or at high risk for COPD, such as COPDGene and SPIROMICS, combined with advances in high-throughput metabolomics now permit the large-scale interrogation of the metabolome in COPD.

A similar integrative approach to large-scale transcriptomics^17^ and proteomics^18^ studies in COPDGene, SPIROMICS, and other cohorts has revealed that a significant amount of variation in many biomarkers is explained by genetic variation, which may also impact metabolome signatures, as recently reported.^19^ In addition, genome-wide association studies (GWASs) have found multiple genetic loci associated with COPD.^20^ Thus, it is important to consider the role of genetic background in assessing how the metabolome relates to COPD; however, to our knowledge, comprehensive studies integrating genetics with comprehensive metabolomic profiling in COPD are lacking. This study identifies plasma metabolites associated with COPD-related phenotypes and addresses the impact of genetic variation on metabolomic profiles in COPD.

## Materials and Methods

### Study populations

#### Discovery

The NIH-sponsored multicenter Genetic Epidemiology of COPD (COPDGene; ClinicalTrials.gov Identifier: NCT01969344) study was approved and reviewed by the institutional review board at all participating centers.^21^ All study participants provided written informed consent. This study enrolled 10,198 non-Hispanic white (NHW) and African American (AA) individuals from January 2008 until April 2011 (Phase 1), who were 45–80 years of age with ≥10 pack-year smoking history and no exacerbation for >30 days. In addition, 465 age- and gender-matched healthy individuals with no history of smoking were enrolled as controls (mostly at Phase 2). From July 2013 to July 2017, 5697 subjects returned for an in-person 5-year visit. Each in-person visit included spirometry before and after albuterol, quantitative computed tomography (CT) imaging of the chest, and blood sampling.

From two clinical centers (National Jewish Health and University of Iowa), 1136 subjects (1040 NHW, 96 AA) participated in an ancillary study in which they provided fresh frozen plasma collected using an 8.5 mL p100 tube (Becton Dickinson) at Phase 2. After excluding AA subjects due to small sample size and subjects lacking genotype data, to avoid confounding genetic associations due to ancestry, 957 subjects comprised the Discovery cohort (Supplementary Fig. S2).

#### COPDGene: Emory

From the Discovery cohort, 271 COPDGene NHW subjects who previously had their metabolome quantified at Phase 2 on a separate platform were used as a technical COPDGene—Emory cohort. This cohort will be referred to as *COPDGene—Emory*.

#### SPIROMICS: Metabolon/UC

Two cohorts from the Subpopulations and Intermediate Outcome Measures in COPD study (SPIROMICS) (ClinicalTrials.gov Identifier: NCT01969344) were used for replication.^22^ The *SPIROMICS—*Metabolon and *SPIROMICS—UC* subjects consisted of 445 and 76 NHW subjects, respectively, who provided fresh frozen plasma using a 10 mL EDTA tube (Becton Dickinson) before a research bronchoscopy.^23^

### Clinical data and definitions

COPD was defined using spirometric evidence of airflow obstruction (post-bronchodilator forced expiratory volume at one second [FEV_1_]/forced vital capacity [FVC] <0.70). PRISm subjects had an FEV_1_ percent predicted (FEV_1_pp) <80% with an FEV_1_/FVC ≥0.7. PRISm subjects have recently been recognized as having a higher prevalence of symptoms and worse outcomes compared to traditionally defined controls,^24^ and were thus included in all cohorts. Chronic bronchitis was defined as self-reported chronic cough and sputum for at least 3 months in each of the 2 years before Phase 2. Percent emphysema was quantified by percent of lung voxels less than −950 Hounsfield Units (% low attenuation areas) on the inspiratory CT scans. Visual emphysema was assessed as previously described.^25^ Exacerbations were defined as acute worsening of respiratory symptoms requiring treatment with oral corticosteroids and/or antibiotics, emergency room visit, or hospital admission.^26^

### Metabolite profiling

#### Discovery platform

P100 plasma was profiled using the Metabolon (Durham) global metabolomics platform, as described.^27–29^ Briefly, samples were extracted with methanol under vigorous shaking for 2 min (Glen Mills GenoGrinder 2000) followed by centrifugation to remove protein, dissociate small molecules bound to protein or trapped in the precipitated protein matrix, and recover chemically diverse metabolites. The resulting extract was divided into five fractions: two for analysis by two separate reverse-phase/ultrahigh-performance liquid chromatography/tandem mass spectrometry (RP/UPLC-MS/MS) methods with positive ion mode electrospray ionization (ESI), one for analysis by RP/UPLC-MS/MS with negative ion mode ESI, one for analysis by hydrophilic interaction liquid chromatography (HILIC)/UPLC-MS/MS with negative ion mode ESI, and one was reserved for backup.

Metabolon has developed peak detection and integration software to generate a list of (mass-to-charge) *m/z* ratios, retention indices (RI), and area under the curve values for each detected metabolite, as described in detail.^27–29^ User-specified criteria for peak detection included thresholds for signal to noise ratio, area, and width. Relative standard deviations of peak area were determined for internal and recovery standards to confirm extraction efficiency, instrument performance, column integrity, chromatography, and mass calibration.

The biological data sets, including quality control samples, were chromatographically aligned based on a retention index that utilized internal standards assigned a fixed RI value. The RI of the experimental peak was determined by assuming a linear fit between flanking RI markers whose RI values are set. Peaks were matched against an in-house library of authentic standards and routinely detected unknown compounds specific to the respective method. Identifications were based on retention index values, experimental precursor mass match to the library authentic standard within 10 ppm, and quality of MS/MS match. All proposed identifications were then manually reviewed and curated by an analyst who approved or rejected each identification based on the criteria above. The platform reported 1392 features, including 1064 annotated features, which were grouped by Metabolon into “super pathways,” including 436 lipids, 261 xenobiotics, 207 amino acids, 40 peptides, 38 cofactors and enzymes, 35 nucleotides, 25 carbohydrates, 11 energy pathway compounds, and 11 partially characterized molecules (Supplementary Table S1). All compounds are further annotated by “subpathway” (e.g., “sphingomyelins,” “carnitine metabolism,” and “lysine metabolism”).

#### COPDGene: Emory

Compounds from p100 fresh frozen plasma were extracted using an untargeted LC-MS-based metabolomic quantification protocol from the laboratory of Dean Jones at Emory University as described previously.^30^ In brief, eight stable isotope internal standards in 130 μL acetonitrile were mixed with 65 μL of plasma. Samples were precipitated and chromatographic separation of the supernatant was performed on a Dionex Ultimate 3000 UHPLC with a dual column compartment for column switching. Reverse phase (C18), anion exchange (AE), and HILIC preceded mass spectral detection using a Thermo Scientific Q-Exactive HF mass spectrometer in continuous full scan mode at 70,000 resolution (scan range 85–1275 *m/z* for all analyses other than AE, AE scan range was 100–1500 *m/z*).

Data were extracted using xMSanalyzer^31^ and annotated using xMSannotator.^32^ There were 4474 features identified among the 271 samples.

#### Spiromics: Metabolon

P100 plasma was profiled using the Metabolon Global Metabolomics Platform, as described for the *Discovery* cohort. The platform reported 1174 features (unannotated features were excluded) with a super pathway breakdown of 435 lipids, 228 amino acids, 318 xenobiotics, 43 cofactors and vitamins, 43 peptides, 41 nucleotides, 30 partially characterized molecules, 25 carbohydrates, and 11 energy metabolites.

#### Spiromics: UC

Samples from p100 fresh frozen plasma underwent LC-MS profiling in the laboratory of Nichole Reisdorph at the University of Colorado Anschutz Medical Campus as previously described.^33,34^ In brief, cold methanol was added to plasma sample aliquots containing internal standards to precipitate proteins. Supernatants were extracted using liquid-liquid extraction with methyl *tert*-butyl ether to obtain a lipid fraction and a small molecule aqueous fraction. Samples were analyzed in positive mode using C18 and HILIC on an Agilent 6545 quadrupole time-of-flight (QTOF) and 6520 QTOF, respectively. Spectral peaks were extracted using MassHunter Profinder B.08 (Agilent). Features were annotated using Mass Profiler Professional (Agilent) using either an in-house accurate mass and retention time (AMRT) database or exact mass and isotope ratios for the compounds that were not in the AMRT database. There were 10,561 features detected among the 81 samples.

### Genotyping

#### Discovery and COPDGene: Emory

Subjects were genotyped using the HumanOmniExpress array (Illumina) employing BeadStudio quality control, which included reclustering on project samples following Illumina guidelines, as previously described for COPDGene. Genotype imputation was performed using the Michigan Imputation Server and the HRC 1.1 reference NHW and the 1000 Genome Phase 1 v3 for AAs.^35^ Ancestry-based principal components (PCs) were calculated and used as previously described.^36,37^ Variants were filtered to include only single-nucleotide polymorphisms (SNPs) with minor allele frequencies >1% in the sample population.

#### Spiromics

Subjects were genotyped using the HumanOmniExpress array (Illumina) as previously described.^38^ Around 683,998 directly genotyped SNPs passed quality control after the removal of SNPs significantly deviating from Hardy-Weinberg expectations (*p*<0.0001), missing allele data (any “0”), and with a genotype call rate <90% and heterozygous haploid genotypes. Genotype imputation was performed using the Michigan imputation server and the HRC 1.1 reference NHW ancestry-based PCs were calculated and used as previously described.^36^ Variants were filtered to include only SNPs with minor allele frequencies >1% in the sample population.

### Statistical analysis

#### Data sets and availability

Clinical data and genotype data can be found on dbGaP for COPDGene (phs000179.v6.p2) and SPIROMICS (phs001119.v1.p1). For COPDGene, the following dataset was used: COPDGene_P1P2_All_Visit_29Sep2018. For SPIROMICS, the CORE 5 data sets were used. *Discovery* metabolomic data are available at the NIH Common Fund's National Metabolomics Data Repository website, the Metabolomics Workbench, https://www.metabolomicsworkbench.org where it has been assigned project ID PR000907.

#### Pre-analyses

##### Discovery and Spiromics: Metabolon

Unless otherwise mentioned, all metabolite data processing and analysis were performed in R (v3.5.1). A data normalization step was performed to correct variation resulting from instrument interday tuning differences: metabolite intensities were divided by the metabolite run day median and then multiplied by the overall metabolite median. It was determined that no further normalization was necessary based on the reduction in the significance of association between the top metabolomics PCs (calculated using the R function “prcomp”) and sample run day after normalization (Supplementary Fig. S3). Metabolites were excluded if >20% of samples were missing values.^39^ For the 995 remaining metabolites, missing values were imputed across metabolites with *k*-nearest neighbor imputation (*k*=10) using the R package “impute.”^40^

To detect and remove outliers, median standard deviation scores (*z*-scores) were calculated across metabolites at the subject level. Subjects with aggregate metabolite median *z*-scores >3.5 standard deviation from the mean (*N*=6) of the cohort were removed (Supplementary Fig. S4). All measured metabolite relative abundances were transformed using the normal quantile transformation, as this type of rank-based transformation can remove possible bias due to outliers or skewed distribution.^41^

##### COPDGene: Emory

Metabolite data were preprocessed using the MSPrep R package.^42^ Data were first imported and summarized across three technical replicates before filtering to include only compounds with <20% missingness over samples. This reduced the data to 2891 compounds, 163 of which were annotated with compound name. Bayesian principal component analysis was employed for imputation^43,44^ of missing values before ComBat batch correction and quantile normal transformation.^45^

##### Spiromics

Metabolite data were pre-processed using the MSPrep R package^42^ as described previously.^33^ Raw data were filtered to include only compounds with <20% missingness over samples. This reduced the data to 7918 compounds, 3843 of which were annotated by compound name. *k*-Nearest neighbor imputation (*k*=5) was employed for imputation of missing values before ComBat batch correction and quantile normal transformation^45^

##### Exploring associations between COPD and metabolites in Discovery cohort

Phenotype-metabolite associations were tested using various regression models and covariates based on previous literature (Supplementary Table S2)^46^ for five phenotypes. Significance was determined within each phenotype at a *p*-value <5.03×10^−5^ after employing a Bonferroni correction to account for multiple testing over 995 metabolites.

##### Metabolome-wide association study

First, the additive effects of SNPs on metabolite abundances were assessed in the Discovery cohort with linear regression using the R package “MatrixEQTL” (version 2.2).^47^ Models were adjusted for clinical covariates (clinical center, sex, age, BMI, smoking pack years, and current smoking status) as well as ancestry-based PCs and as previously described.^36^ Metabolite quantitative trait loci (met-QTLs) were considered significant at *p*-value <6.6×10^−12^ for genome-wide significance after employing a Bonferroni correction to account for multiple testing across 995 metabolites and 7,641,295 genotyped and imputed SNPs.

##### Metabolome-wide association study replication across metabolomic platforms and cohorts

Significant met-QTL SNPs were tested for associations in the COPDGene—Emory and SPIROMICS replication platforms, using the same methods as previously described for the Discovery cohort and the Bonferroni correction for multiple testing.

##### Recursive conditioning

If *K* met-QTL-SNPs were associated with a metabolite with *p*-values smaller than 6.6×10^−12^, *p*-values were calculated for each of the *K*−1 SNPs conditioning on the top SNP identified in the met-QTL analysis and other covariates (age, sex, BMI, smoking status, smoking pack-years, and clinical center). The SNP with the smallest *p*-value was considered an independent met-QTL if *p*-value <0.05/(*K*−1), where 0.05/(*K*−1) was the *p*-value threshold by Bonferroni correction. We applied this procedure iteratively until the smallest *p*-value was larger than 0.05/*T*, where *T* is the number of remaining SNPs.^36^

##### Exploring met-QTLs

Percent variance explained by SNPs and clinical variables was calculated using the coefficient of determination (*r*^2^). met-QTL features were characterized using the “—most_severe_variant” filter and nearest genes were identified using the “—nearest symbol” argument in the Ensembl Variant Effect Predictor (VEP) tool (V97).^48^

##### Enrichment analysis

Group enrichment (i.e., subpathway for metabolites or variant class for SNPs) among significantly associated features was statistically assessed against the entire feature set using a one-tailed Fisher's exact test.^49^ Results were adjusted using Benjamini and Hochberg^50^ (a.k.a. false discovery rate) with an alpha of 0.05.

##### Network analysis of metabolic interaction

As metabolic pathway annotations are arbitrarily defined and ignore unannotated compounds,^51,52^ we sought to identify COPD-affected pathways in a strictly data-driven manner. This was performed in a two-step procedure. First, we generated a Gaussian graphical model (GGM) of metabolite co-abundance based on partial correlation coefficients corrected for the effects of all other metabolites and potential confounders (age, sex, BMI, smoking status, smoking pack-years, and clinical center).^53^

The use of partial coefficients in the GGM model seeks to overcome a major drawback of other correlation networks (e.g., Pearson's) by conditioning against correlations with all other variables. Edges between metabolites were present if partial correlations were statistically significant at an alpha of 0.05, after Bonferroni correcting for $\left(\begin{array}{c} 995\\ 2 \end{array}\right)$ tests, with a positive partial correlation >0.2 to declare whether an edge is “present” in the network view.

Negative partial correlations likely represent spurious signals as detailed in previous publications,^53,54^ and thus were removed. To infer potential genetic effects, results from the metabolome-wide association study (mWAS) were included in the network view by introducing “SNP” nodes with edges present between met-QTLs and associated metabolites.^54^ In summary, the combined GGM and mWAS approach will provide an unbiased map of metabolic pathways and their genetic influences.^53^

The first step based on the GGM identifies partially correlated metabolites, but does not consider phenotypes. Therefore, in the second step, metabolomic modules associated with COPD phenotypes were identified using a greedy search algorithm.^55^ Each phenotype was tested separately. Briefly, each metabolite node was regressed against the phenotype and scored using the negative logarithmized *p*-value of the phenotype beta coefficient. Phenotypes were adjusted for the same covariates as identified in previous literature (Supplementary Table S2)^46^ by regressing the phenotype against those covariates and using the residuals as the independent variable in the model. Next, starting with a seed node, each neighboring node is added iteratively to the candidate module by averaging metabolite intensities, and this extended module is scored by linear regression as previously described. The neighbor is added only if the score of the newly extended module is higher than the scores of all the single components. Any overlapping optimal module is combined in a final step into a single module and scored by the scoring function, using the same rules as before to determine inclusion.

In summary, this approach systematically identifies phenotype-affected modules based on a GGM-derived network of metabolic pathways. Both steps were performed using the R package “MoDentify”^55^ and visualized using Cytoscape (v3.71).^56^

## Results

### Metabolome data substructure

Before reducing data by the exclusion criteria, we first explored the metabolomic profiles of all COPDGene subjects with metabolomes quantified by Metabolon at Phase 2. These subjects were representative of all COPDGene subjects who returned for the 5-year follow-up (Supplementary Table S3). Pairwise correlations among metabolites were assessed using Pearson's *r* for hierarchical clustering within Metabolon-defined super pathways. Beyond a positively correlated cluster of lipids, metabolites exhibited minor correlation (Supplementary Fig. S5).

Univariate demographic associations were then assessed with linear regression models. The demographic variables most strongly associated with metabolites included age, sex, race, and smoking status (Supplementary Table S4 and Supplementary Fig. S5). Of the 995 metabolites tested, 398 (40.2%) were significantly associated with age, 319 (32.1%) with sex, 355 (37.2%) with race, 250 with BMI (25.1%), and 128 (12.9%) with smoking status. Enrichment analysis found androgenic steroids, acylcarnitines, and dicarboxylates to be enriched for associations with age; sphingomyelin, androgenic steroids, and phosphatidylcholines with sex; xanthines and dicarboxylates with race; and diacylglycerols and branched chain amino acids (BCAAs) for BMI (Supplementary Table S5).

### Study subjects

Demographic and clinical characteristics of the Discovery cohort are shown in Table 1. There were significant differences between PRISm subjects, current or former smoker controls, and COPD across age, sex, BMI, smoking status, and smoking pack-years. Among the met-QTL replication cohorts, COPDGene—Emory subjects were representative of the Discovery cohort, while the SPIROMICS subjects were slightly younger and healthier, as evidenced by the higher FEV_1_pp and lower percent emphysema (Table 2).

**Table 1.** Demographics of Discovery cohort

	Total	PRISm	Control	COPD	Missing	p
No. of participants (%)	957	85 (8.9)	390 (40.8)	468 (48.9)	14 (1.4)	
Age^a^	68.3 (8.4)	66.7 (7.3)	65.9 (8.5)	70.5 (8.0)	70.7 (5.8)	<0.0001
Male sex (%)	490 (51.2)	31 (36.5)	184 (47.2)	268 (57.3)	7 (50.0)	0.0002
BMI (%)	29.1 (6.2)	32.6 (7.7)	29.3 (5.6)	28.2 (6.1)	27.9 (5.3)	<0.0001
Current smoker (%)	204 (21.3)	24 (28.2)	78 (20.0)	98 (20.9)	4 (28.6)	0.1914
Smoking pack-years^a^	46.0 (24.9)	48.6 (24.3)	36.1 (19.5)	53.6 (26.2)	51.7 (22.2)	<0.001
FEV_1_pp_utah^a^	76.6 (26.5)	70.2 (7.4)	99.2 (11.5)	58.9 (23.5)	NA	NA
FEV_1_/FVC^a^	0.7 (0.2)	0.8 (0.0)	0.8 (0.0)	0.5 (0.1)	NA	NA
Percent emphysema^a^	7.3 (10.2)	1.6 (2.5)	2.2 (2.6)	12.9 (12.2)	9.2 (11.7)	<0.0001

Chi-square tests were used to test for differences between groups in binary variables. One-way ANOVA tests were performed to test for differences between groups in continuous variables.

PRISm, Preserved Ratio Impaired Spirometry^23^; COPD is defined by GOLD score ≥1; missing, 14 subjects were deemed ineligible for spirometry and thus did not have a defined GOLD status. These subjects were still included in analyses with other COPD phenotypes and the met-QTL analysis.

BMI, body mass index (kg/m^2^); FEV_1_/FVC, post-bronchodilator forced expiratory volume at one second/forced vital capacity; FEV_1_pp, FEV_1_ percent predicted.

^a^ Mean and standard deviations provided.

COPD, chronic obstructive pulmonary disease.

**Table 2.** Demographics of replication cohorts

	COPDGene—Emory	SPIROMICS—Metabolon	SPIROMICS—UC	p
No. of participants	271	445	76	NA
Age^a^	67.3 (8.4)	65.3 (8)	61.6 (8)	<0.001
Male sex (%)	127 (46.9)	244 (54.8)	40 (52.6)	0.1164
BMI (%)	28.7 (5.8)	28.1 (4.9)	28.3 (4.8)	0.3675
Current smoker (%)	57 (21.0)	116 (26.4)	19 (25.7)	0.2665
Smoking pack-years^a^	43.9 (23.3)	47.8 (31.6)	43.2 (24)	0.1362
FEV_1_pp_utah^a^	77.1 (25.3)	79.4 (23.5)	89.3 (20.8)	0.0004
FEV_1_/FVC^a^	0.7 (0.1)	0.6 (0.1)	0.7 (0.1)	0.0015
Percent emphysema^a^	6.9 (9.7)	6.1 (8.2)	4 (4.8)	0.038

Chi-square tests were used to test for differences between groups in binary variables. One-way ANOVA tests were performed to test for differences between groups in continuous variables.

^a^ Mean and standard deviation provided unless otherwise noted.

### Metabolites associated with COPD phenotypes in the Discovery cohort

**FIG. 1.**
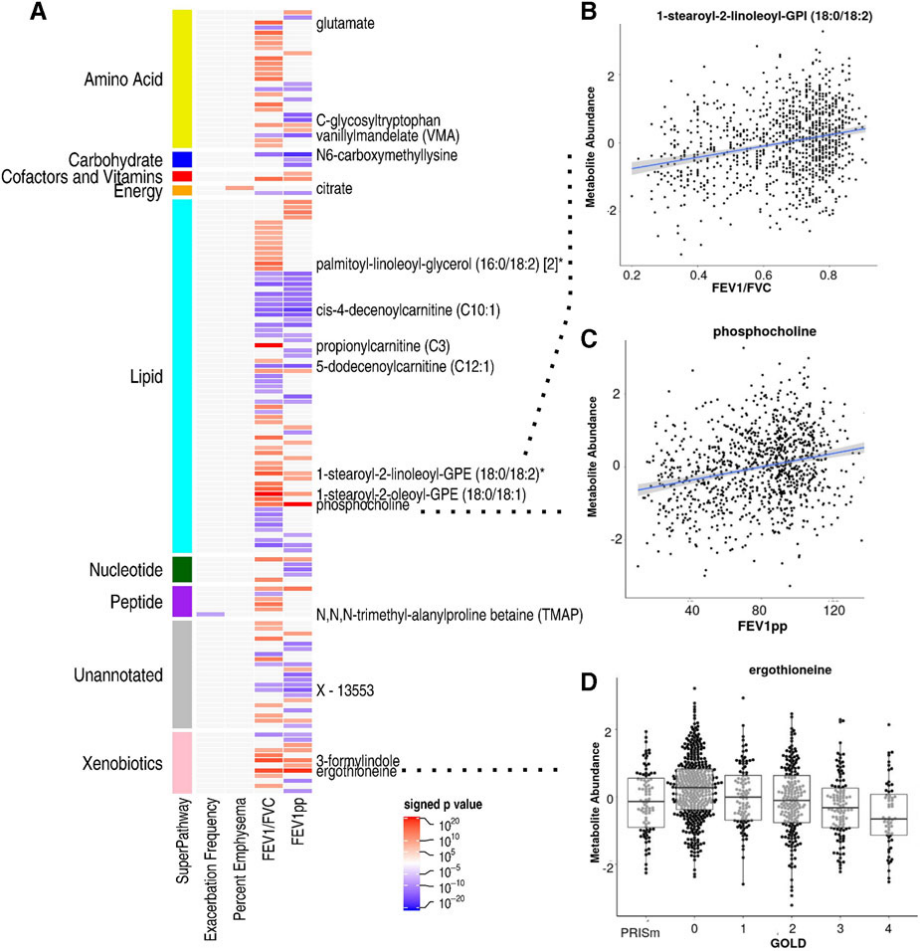
Metabolite associations with COPD. **(A)** Heat map of signed *p*-values. Metabolites are organized by super pathway. Red intensity indicates positive direction of effect, while blue intensity indicates negative. Only select metabolites most significantly associated are labeled. **(B)** Scatter plot of 1-stearoyl-2-linoleoyl-GPI (18:0/18:2) abundance by FEV_1_/FVC ratio. **(C)** Scatter plot of phosphocholine abundance by FEV_1_pp. (**D)** Bee swarm of ergothioneine abundance by GOLD stage. Ergothioneine was one of the topmost associated metabolites with all airflow obstruction phenotypes. Metabolites are color coded by Super Pathway designation. Metabolite abundances are inverse normal transformed. *Indicates compounds that have not been officially confirmed based on a standard, but Metabolon is confident in its identity. COPD, chronic obstructive pulmonary disease; FEV_1_, forced expiratory volume at one second; FEV_1_pp, FEV_1_ percent predicted; FVC, forced vital capacity; GPI, glycophosphatidylinositol.

Of the 995 metabolites tested for associations, 147 (14.8%) were significantly associated with at least 1 of the 5 COPD phenotypes studied (Fig. 1A, full results in Supplementary Tables S6–S10). There was no metabolite significantly associated with chronic bronchitis. For exacerbations and emphysema, only one metabolite was identified in each. Higher abundance of *N*,*N*,*N*-trimethyl-alanylproline betaine (TMAP) was significantly associated with a decrease in exacerbation frequency (*p*=3.75×10^−5^), while increased abundance in a tricarboxylic cycle metabolite (citrate) was significantly associated with higher percent emphysema (*p*=5.2×10^−6^).

For the COPD phenotypes characterized by airflow obstruction, 145 metabolites from 55 subpathways were significantly associated with either FEV_1_pp or FEV_1_/FVC. For FEV_1_/FVC, there were significant associations with 99 metabolites from 30 subclasses, 39 (39.4%) of which were positively associated (Fig. 1B). Glycophosphatidylinositol (Fig. 1C), propionylcarnitine (C3), and ergothioneine, a xenobiotic (Fig. 1E), were most strongly associated (*p*=2.57×10^−13^, 4.8×10^−13^, and 4.1×10^−11^, respectively). Enrichment analysis found metabolites in the diacylglycerol and BCAA (leucine, isoleucine, and valine) subpathways to be enriched for associations with FEV_1_/FVC (Supplementary Table S11). For FEV_1_pp, 79 metabolites from 23 subclasses were significantly associated, with lipid phosphocholine (Fig. 1D), ergothioneine, and carbohydrate *N*6-carboxymethyllysine most significantly associated (*p*=3.3×10^−13^, 3.28×10^−12^, and 1.4×10^−11^, respectively).

### Identification of SNPs associated with metabolites

**FIG. 2.**
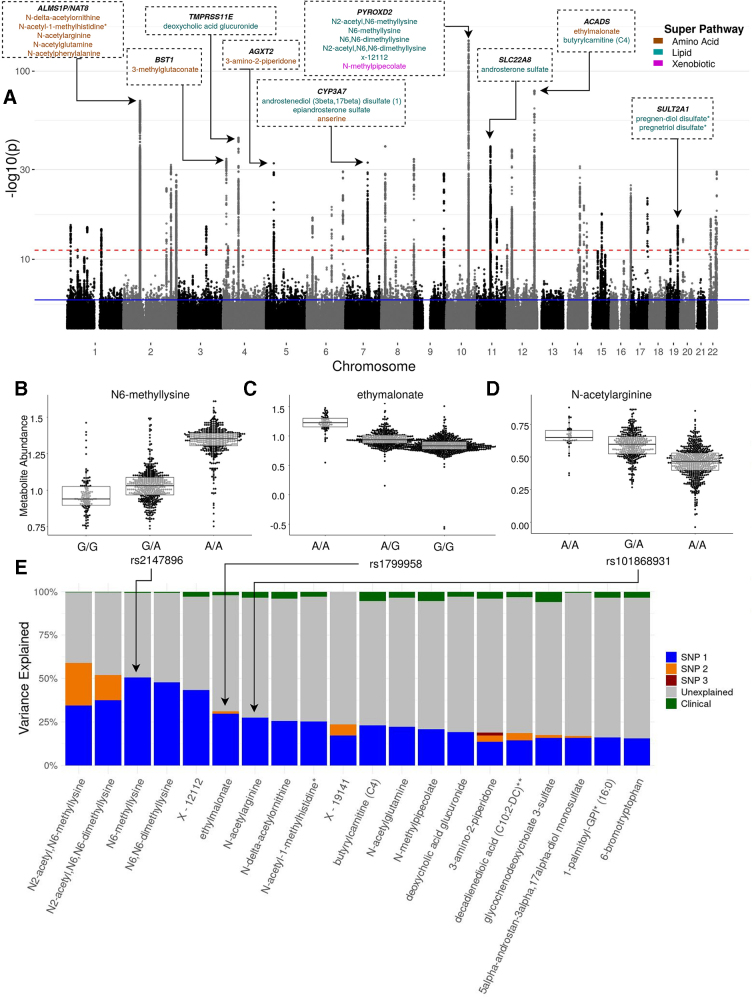
Genome-wide associations between SNPs and metabolites. **(A)** *Discovery mWAS* Manhattan plot showing −log10 *p*-values from mWAS tests. The blue and red dashed lines indicate false discovery rate and Bonferroni significance, respectively. Loci in which >20% of the metabolite variance is explained by a single SNP are labeled by nearest gene and metabolites affected. Metabolite text colors coded by Super Pathway. **(B–D)** Bee swarms of inverse normal transformed metabolite abundances by genotype for metabolites of different subpathways with the greatest variance explained by one SNP. Overlayed box plots represent the median and interquartile range of transformed metabolite abundance. **(E)** Bar plot of the percent variation for metabolites explained by clinical (green), top mQTL SNP (blue), the second independent mQTL SNP (orange), and any more independent mQTL SNPs (red). The gray indicates unknown variance. Clinical factors include age, sex, BMI, smoking status, smoking pack-years, and clinical center. BMI, body mass index; mWAS, metabolome-wide association study; SNPs, single-nucleotide polymorphisms.

We next investigated the genetic contribution to metabolite abundances by investigating the relationship between genotypes and metabolites. Of the ∼7.6 million genotyped and imputed SNPs tested, we identified 4281 met-QTL SNPs associated with 109 (10.95%) of metabolites tested in the Discovery cohort (Fig. 2A and Supplementary Table S12). An interactive plot displaying met-QTL SNP association with metabolite subclass can be found at https://plot.ly/∼lagillenwater/7 Using recursive conditioning, 79 independent SNPs were identified with an additive relationship with the 109 metabolites (Supplementary Table S13 and Fig. 2C, D). At least 15% of the variance in 20 metabolites was explained by 1 or more of these SNPs, often much more than observed in clinical variables (Table 3 and Fig. 2E).

**Table 3.** Metabolite single-nucleotide polymorphisms explaining >15% of the variation in blood

Metabolite	Super pathway	Subpathway	Variance explained	rsID	Consequence	Closest gene	Effect allele	Other allele	EAF	p
*N*6-methyllysine	Amino acid	Lysine metabolism	0.50	rs2147896	Missense	*PYROXD2*	A	G	0.64	2.68×10^−146^
*N*6,*N*6-dimethyllysine	Amino acid	Lysine metabolism	0.48	rs2147896	Missense	*PYROXD2*	A	G	0.64	1.98×10^−135^
X - 12112	Unannotated	Unannotated	0.44	rs7905265	Downstream gene	*PYROXD2*	C	G	0.64	6.00×10^−119^
*N*2-acetyl,*N*6,*N*6-dimethyllysine	Amino acid	Lysine metabolism	0.38	rs10182082	Intron	*NAT8*	G	C	0.22	6.07×10^−38^
			0.15	rs7905265	Downstream gene	*PYROXD2*	C	G	0.64	1.86×10^−97^
*N*2-acetyl,*N*6-methyllysine	Amino acid	Lysine metabolism	0.34	rs7905265	Downstream gene	*PYROXD2*	C	G	0.64	5.04×10^−88^
			0.25	rs10182082	Intron	*NAT8*	G	C	0.22	6.51×10^−65^
Ethylmalonate	Amino acid	Leucine, isoleucine and valine metabolism	0.30	rs1799958	Missense	*ACADS*	A	G	0.25	5.87×10^−79^
			0.01	rs6490297	Intergenic	*CABP1*	C	T	0.27	9.09×10^−28^
*N*-acetylarginine	Amino acid	Urea cycle; arginine and proline metabolism	0.27	rs10168931	Intron	*NAT8*	A	G	0.23	5.63×10^−70^
*N*-delta-acetylornithine	Amino acid	Urea cycle; arginine and proline metabolism	0.26	rs10168931	Intron	*NAT8*	G	A	0.77	2.02×10^−64^
*N*-acetyl-l-methylhistidine^*^	Amino acid	Histidine metabolism	0.25	rs10206899	Intron	*NAT8*	C	T	0.23	1.76×10^−66^
Butyrylcarnitine (C4)	Lipid	Fatty acid metabolism (also BCAA metabolism)	0.23	rs1799958	Missense	*ACADS*	A	G	0.25	1.75×10^−59^
*N*-acetylglutamine	Lipid	Primary bile acid metabolism	0.22	rs4149056	Missense	*SLCO1B1*	G	T	0.16	4.51×10^−39^
*N*-methylpipecolate	Xenobiotics	Bacterial/Fungal	0.21	rs2147896	Missense	*PYROXD2*	A	G	0.64	6.75×10^−54^
Deoxycholic acid glucuronide	Lipid	Secondary bile acid metabolism	0.19	rs34594059	Intron	*TMPRSS11E*	C	C	0.36	5.79×10^−45^
X - 19141	Unannotated	Unannotated	0.17	rs34436963	3′ UTR	*TMPRSS11E*	G	A	0.64	2.12×10^−43^
			0.06	rs1165196	Missense	*SLC17A1*	G	A	0.45	3.08×10^−17^
1-Palmitoyl-GPI^*^ (16:0)	Lipid	Lysophospholipid	0.16	rs102275	Intron	*TMEM258*	T	C	0.64	2.38×10^−41^
5alpha-androstan-3alpha,17alpha-diol monosulfate	Lipid	Androgenic steroid	0.16	rs1495741	Intergenic	*NAT2*	G	A	0.23	4.41×10 ^−41^
			0.01	rs1041983	Synonymous	*NAT2*	C	T	0.67	6.28×10^−15^
Glycochenodeoxycholate 3-sulfate	Lipid	Primary bile metabolism	0.16	rs4149056	Missense	*SLCO1B1*	T	T	0.16	4.51×10^−39^
			0.01	rs11045913	Downstream gene	*SLCO1A2*	G	A	0.56	3.07×10^−15^
Decadienedioic acid (C10:2-DC)^**^	Lipid	Fatty acid, dicarboxylate	0.14	rs11621061	Intron	*HEATR4*	C	T	0.76	1.89×10^−28^
			0.04	rs58231493	Upstream gene	*ACOT2*	C	T	0.56	8.80×10^−32^
3-Amino-2-piperidone	Amino acid	Urea cycle; arginine and proline metabolism	0.13	rs37369	Missense	*AGXT2*	T	C	0.08	6.06×10^−33^
			0.04	rs16899974	Missense	*AGXT2*	A	C	0.22	9.31×10^−17^

Metabolite, metabolite annotation; super pathway, metabolite class annotation; subpathway, within class pathway annotation; variance explained, metabolite variance explained by genotype variation in mQTL SNP.

Total variance explained by mQTLs—metabolite variance explained by variation in all mQTL SNPs.

*p*-Value, *p*-value of regression test with SNP.

^*^ Indicates compounds that have not been officially confirmed based on a standard, but Metabolon is confident in its identity.

^**^ Indicates a compound for which a standard is not available, but Metabolon is confident in its identity or the information provided.

(#) or [#] indicates a compound that is a structural isomer of another compound in the Metabolon spectral library.

Ancestral allele, major allele; closest gene, closest gene to SNP as mapped in VEP; consequence, VEP annotation of variant; EAF, effect allele frequency in Discovery; effect allele, allele with positive association to metabolite; rsID, reference SNP ID number assigned by NCBI.

BCAA, branched chain amino acid; GPI, glycophosphatidylinositol; SNP, single-nucleotide polymorphism; VEP, Variant Effect Predictor.

The strongest genetic link was between a missense variant in the *PYROXD2* region of chromosome 10, rs2147896, which explained 50.48% of the variance of *N*6-methyllysine; in contrast, the clinical variables explained only 0.64% of the variance in this metabolite (Fig. 2E). For 13 metabolites, 2 or more independent met-QTL SNPs contribute to metabolite variance (Table 3). For example, 58.90% of variance in *N*2-acteyl, *N*6-methyllysine is explained by variants in *PYROXD2* (34.26%) and *NAT8* (24.64%) regions.

### Biologic significance of met-QTL SNPs and associated metabolites

Next, we set out to determine if these met-QTL SNPs have been previously associated with COPD, lung function, or metabolite levels. First, we cross-referenced the 4281 variants with 279 significant SNPs from a recent lung function GWAS^57^ and164 reported primary and secondary COPD GWAS SNPs^58^ for overlapping associations. Next, we compared the met-QTL variants with published associations in the NHGRI GWAS catalog.^59^ Of the expanded variant set, 351 SNPs have been previously reported, mostly in other metabolomic analyses.^54,60–63^ There were seven met-QTL SNPs that had previously been associated with smoking habits (SNPs rs10254729, rs10469966, rs12825376, rs13437771, rs2072113, rs2421667, and rs883403), although no met-QTL SNP overlapped with lung function or COPD GWAS SNPs.

**FIG. 3.**
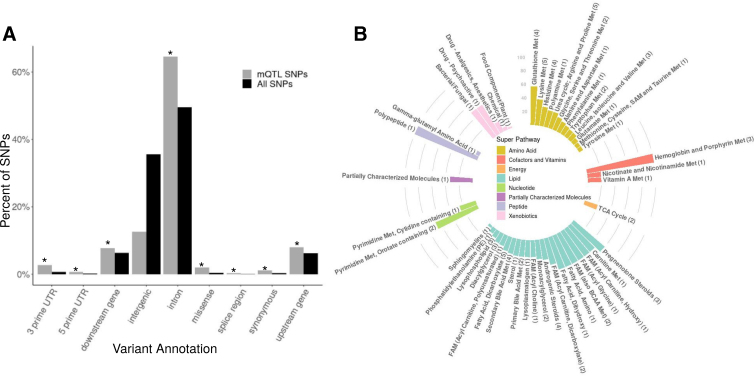
mQTL SNP enrichment analyses. **(A)** A bar plot showing the percentage by variant annotation of mQTL SNPs (black) and all SNPs tested (black). *Variant annotations significantly enriched in mQTLs. **(B)** A circular bar plot showing the percentage of each subpathway with at least one independent mQTL. The bars are colored by super pathway and labeled by subpathway, with the total number of metabolites in the subpathway with an mQTL in parentheses. FAM, fatty acid metabolism; Met, metabolism.

Using Ensembl VEP, we found intronic SNPs to be the most represented met-QTL SNP class (64.5%), followed by intergenic variants (12.6%) (Supplementary Table S14 and Fig. 3A). Intronic variants were also the most significantly enriched, followed by 3′ untranslated region and missense variants (*q*=3.48×10^−79^, 3.99×10^−33^, and 2.68×10^−25^). At least 50% of metabolites in 13 subpathways had met-QTLs, with all 3 of the metabolites in the hemoglobin and porphyrin metabolism subpathway having significant genetic associations (Supplementary Table S15 and Fig. 3B). Although metabolites with met-QTLs were not enriched for any subpathway, at the super-pathway level, an enrichment of amino acids was found (*q*=0.048).

### Replication of met-QTL SNPs

**FIG. 4.**
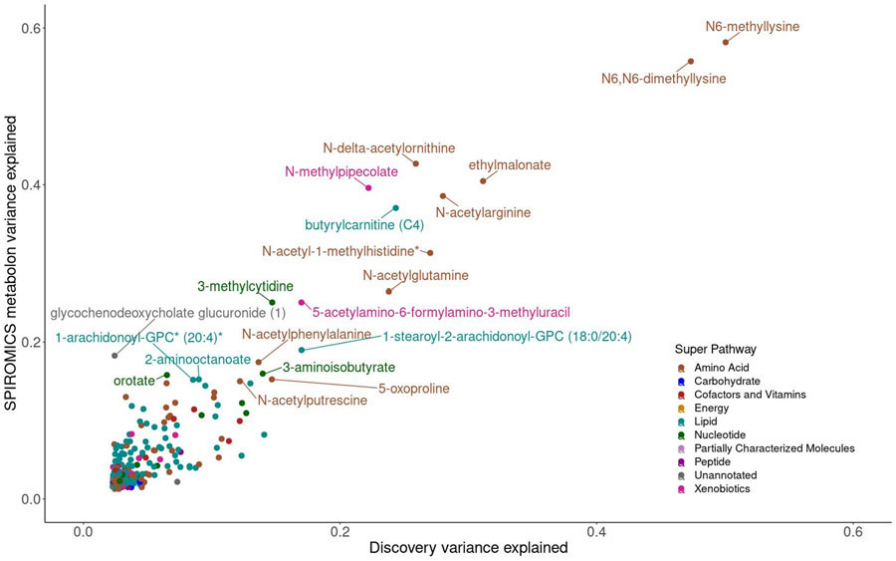
Scatter plot of metabolite variance explained by lead SNP in Discovery and SPIROMICS—Metabolon cohorts. We calculated the variance explained (*r*^2^) for each lead mQTL SNP and metabolite by cohort. Metabolite colors represent super pathway annotation and are labeled if variance explained by genotype was >0.15 in either cohort.

We used three strategies to test for replication of met-QTL SNPs (see Materials and Methods section). First, we used an independent high-resolution LC-MS strategy in a different laboratory in the same cohort (COPDGene—Emory) (Supplementary Table S16). Second, we used an independent cohort with data also quantified by Metabolon (SPIROMICS—Metabolon) (Supplementary Table S17). Third, we used an independent cohort with data from an independent platform (SPIROMICS—UC) (Supplementary Table S18). The cohort with the greatest replication was SPIROMICS—Metabolon where 72 met-QTL associations replicated (Fig. 4, Table 4, and Supplementary Fig. S6), with similar metabolomic variance explained. Replications were seen for several chromosomal regions across Discovery, COPDGene—Emory, and SPIROMICS—Metabolon, including lysine metabolites with SNPs in *PYROXD2* on chromosome 10, phosphocholines with SNPs in *MYRF, THEM25B, and FADS1/2* regions on chromosome 11, cysteinylglycine disulfide with *SNPs in DPEP,* and bilirubin/biliverdin with SNPs in the *UGT1A1/8* regions on chromosome 2, with strong signals in the *PYROXD2* of chromosome 10, as well as the *FADS1*/*FADS2* region of chromosome 11 (Table 3 and Supplementary Fig. S5).

**Table 4.** mQTL replications

SNPs	CHR	Closest gene(s)	Discovery	COPDGene—Emory^a^		SPIROMICS—Metabolon
rs2147896, rs7905265	10	*PYROXD2*	***N*2-acetyl,*N*6-methyllysine, *N*-methylpipecolate, *N*6,*N*6-dimethyllysine, *N*6,*N*6,*N*6-trimethyllysine, *N*2-acetyl,*N*6,*N*6-dimethyllysine, *N*6-methyllysine,** X - 12112	506.130672493465_285.4155141		***N*-acetyl-isoputreanine, *N*6-methyllysine, *N*2-acetyl,*N*6-methyllysine, *N*2-acetyl,*N*6,*N*6-dimethyllysine, *N*6,*N*6,*N*6-trimethyllysine, *N*6,*N*6-dimethyllysine, *N*-methylpipecolate**
rs174533, rs102275, rs10224, rs1404372161, rs174567, rs1535	11	*MYRF, THEM25B, FADS2, FADS1*	**1-Palmitoyl-2-arachidonoyl-GPC (16:0/20:4n6)**, Oleoyl-arachidonoyl-glycerol (18:1/20:4) [2],^*^ 1-arachidonoyl-GPC^*^ (20:4),^*^ Oleoyl-arachidonoyl-glycerol (18:1/20:4) [1],^*^ Stearoyl-arachidonoyl-glycerol (18:0/20:4) [1],^*^1,2-dilinoleoyl-GPC (18:2/18:2), 1-palmitoyl-2-dihomo-linolenoyl-GPC (16:0/20:3n3 or 6),^*^1-(1-enyl-palmitoyl)-2-arachidonoyl-GPC (P-16:0/20:4),^*^ 1-stearoyl-2-arachidonoyl-GPC (18:0/20:4)	1222.85903685705_175.407391, 1589.12673654366_174.9453186, 782.570331871481_178.6310904, 1588.12181759391_175.0049822, 1210.34978948251_175.6062675, 872.538808473057_175.3542714, 831.571639521416_172.2185806, 942.54035577298_179.744523, 1617.16142714192_173.1058804, 875.556039922196_181.877006, 1618.17099209145_172.9865179, 1196.33733718313_175.4123396, 1591.13917344304_174.7156649, 1208.8420355579_175.0746202, 1209.35026653248_175.0413014, 1222.35055488247_174.4656717, 874.553705272313_181.378412,	PE(20:3(8Z11Z14Z)18:1(11Z)); PC(15:020:4(8Z11Z14Z17Z)); 1621.20035693998_171.6141601, 1590.14303149284_177.2413577, 783.572309421383_174.840046, 811.603130219842_173.9750369, 1223.36030483198_172.6915558	1-stearoyl-2-linoleoyl-GPI (18:0/18:2), 1-palmitoyl-2-dihomo-linolenoyl-GPC (16:0/20:3n3 or 6),^*^ 1-stearoyl-2-linoleoyl-GPE (18:0/18:2),^*^ 1-linoleoyl-2-linolenoyl-GPC (18:2/18:3),^*^ 1-(1-enyl-palmitoyl)-2-arachidonoyl-GPC (P-16:0/20:4),^*^ 1-oleoyl-2-linoleoyl-GPE (18:1/18:2),^*^ 1-palmitoyl-2-linoleoyl-GPE (16:0/18:2), 1-linoleoyl-GPE (18:2),^*^ 1-(1-enyl-stearoyl)-2-arachidonoyl-GPE (P-18:0/20:4),^*^1-palmitoyl-2-arachidonoyl-GPC (16:0/20:4n6), 1-stearoyl-2-linoleoyl-GPC (18:0/18:2),^*^ 1-arachidonoyl-GPE (20:4n6),^*^ 1-palmitoyl-2-linoleoyl-GPC (16:0/18:2), Linoleoyl-arachidonoyl-glycerol (18:2/20:4) [2],^*^1-arachidonoyl-GPC^*^(20:4),^*^ hydroxypalmitoyl sphingomyelin (d18:1/16:0(OH)), **1-stearoyl-2-arachidonoyl-GPC (18:0/20:4)**, 1-palmitoleoyl-2-linolenoyl-GPC (16:1/18:3),^*^1,2-dilinoleoyl-GPC (18:2/18:2), 1-palmitoyl-2-linoleoyl-GPI (16:0/18:2), linoleoyl-arachidonoyl-glycerol (18:2/20:4) [1]^*^
rs409170, rs1126464	16	*DPEP1*	Cys-gly, oxidized, cysteinylglycine disulfide^*^	**l-Cysteinylglycine disulfide**		cys-gly, oxidized, cysteinylglycine disulfide^*^
rs887829, rs4148324	2	*UGT1A1, UGT1A8*	X - 24849, X - 16946, X - 21448, X - 11522, Succinimide, **Biliverdin**, X - 11530, bilirubin (E,Z or Z,E),^*^bilirubin (E,E),^*^bilirubin	**Biliverdin, bilirubin,** 665.245399337728_54.33459064, 633.255282337234_52.90499415, 285.123456943826_51.407805, 197.107343344632_52.0355726, 581.24057883797_54.58074146, 178.06518309674_51.11991256, 193.076070846196_49.00901634, 209.107371044631_48.9573111, 194.096443195177_49.11705413,	167.072710946364_51.47896251, 191.072722146363_54.49274426, 180.080778195961_51.36922788, 283.10777804461_53.32993737, 299.139042143047_56.91349852, 586.274358886281_65.12241616, 2-(3-Carboxy-3-(methylammonio)propyl)-l-histidine	Bilirubin (E,E),^*^bilirubin, bilirubin (E,Z or Z,E),^*^biliverdin

Bold text indicates replication across cohorts.

^a^ COPDGene—Emory metabolites not annotated by xMSAnnotator are reported as mass-to-charge ratio and retention time.

^*^ Indicates compounds that have not been officially confirmed based on a standard, but Metabolon is confident in its identity.

(#) or [#] indicates a compound that is a structural isomer of another compound in the Metabolon spectral library.

### Integration of genes, metabolites, and COPD phenotypes

The met-QTL analysis provides evidence of genetic-metabolite abundance links, but not specific to COPD. To identify affected genetic-metabolite-phenotype pathways in a strictly data-driven manner, we first created a GGM network of co-abundant metabolites and then used those results to identify modules associated with disease phenotypes. This method has been shown to enhance classical association analyses by increasing statistical power through aggregating metabolite abundance and recognizing disease-driven interplay between pathways.^55^ To infer the relationship between the genomics, metabolomics, and phenotypic data, the mWAS results were combined with phenotype-driven modules by adding edges between met-QTL SNPs and metabolites.

In the first step, given all 995 metabolites, a GGM was created. Then, nodes representing independent met-QTL SNPs were added, with edges linking to associated metabolites. The final GGM network was sparse, containing a combined 693 nodes (582 metabolite and 79 SNP nodes) and 505 significant, undirected edges between any node (metabolites or genes).

**FIG. 5.**
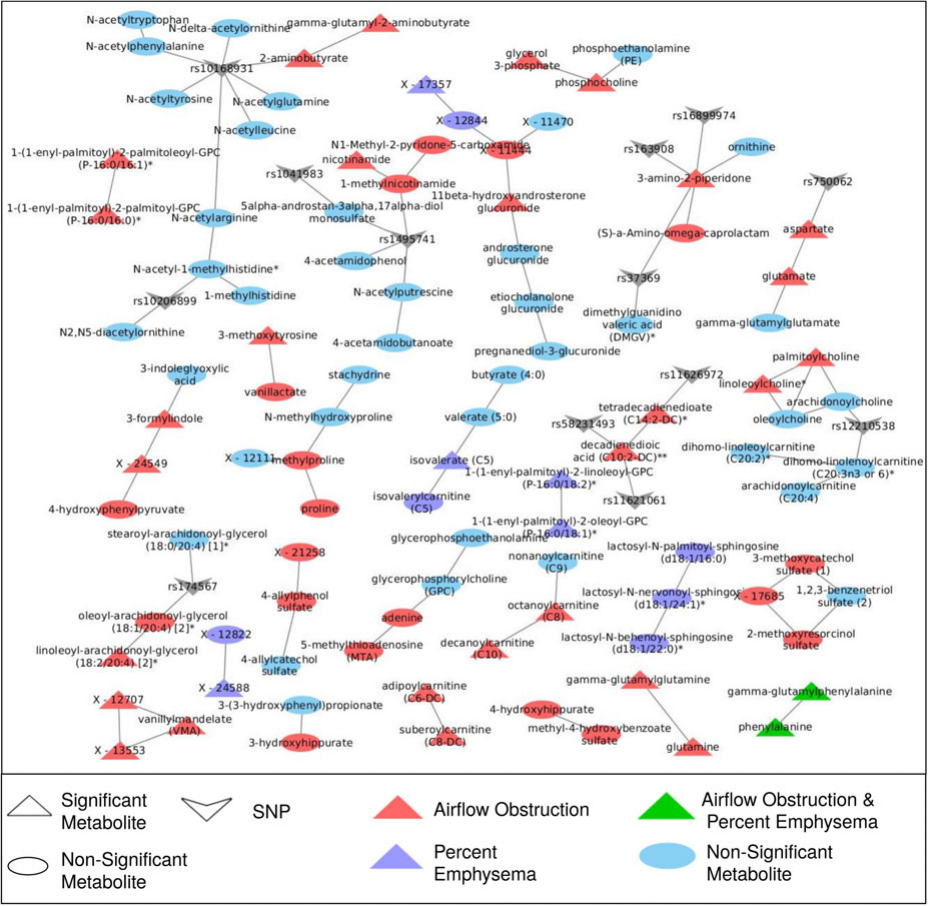
Phenotype-driven modules. Cytoscape network representation of metabolite modules significantly associated one or more COPD phenotypes. Circular nodes are nonsignificant, while triangular nodes were significant in univariate analysis. “V” nodes mQTL SNPs. The color corresponds to the phenotype with which the metabolites in the module are associated; red indicates airflow obstruction phenotypes (FEV_1_/FVC or FEV_1_pp), purple indicates percent emphysema, green indicates both spirometric phenotypes and percent emphysema, and blue are nonsignificant metabolites.

Then, in the second step, we used the *MoDentfy* module-identification algorithm with the COPD phenotypes to find phenotype-associated modules (i.e., subnetworks of the GGM associated with a specific phenotype). Testing all 5 COPD phenotypes separately, this resulted in 26 significant modules, sometimes associated with more than 1 phenotype, which included metabolites missed in univariate analysis (Fig. 5 and Supplementary Table S19). For example, a module of three lactosylceramides was associated with percent emphysema (adjusted *p*-value=0.00018) and a module of three hippurates was associated with FEV_1_/FVC (adjusted *p*-value=0.04), all of which had not been significantly associated previously (although the same direction of effect was observed between the modules and independent metabolites; see Supplementary Table S19). Other modules reconfirmed previously identified associations, like the module most associated with FEV_1_pp (Bonferroni adjusted *p*-value=3×10^−7^) containing the amino acid vanillylmandelate and two unannotated metabolites (X - 12707 and X - 13553).

Of the 26 modules associated with COPD phenotypes, 6 associated with airflow obstruction phenotypes had edges to gene nodes (Table 5). For these modules, we utilized the percent-variance-explained results to determine the genetic effect on individual metabolite abundances and the module, represented by the first PC of the module. We further compared the variance explained by genetic variance to the percent explained by clinical and environmental variance, as represented by the covariates used previously. While, as reported earlier, significant variance in individual metabolites was explained by genetic variance (ranging from 5% to 18.9%), the opposite effect was observed in COPD phenotype-associated modules, with the exception of the module of dicarboxylate fatty acids containing decadienedioic acid (C10:2-DC)** and tetradecadienedioate (C14:2-DC)* where 13.2% of the module variance was explained by variation in SNPs rs11626972 and rs58231493 and only 3% was explained by clinical and environmental variance.

**Table 5.** Module variance explained by genetic and environmental variables

Phenotype	Metabolite	Module ID	Module beta	Adjusted score	Most significant independent SNP	Consequence	Closest gene	Module first PC variance explained by genetic variants	Module first PC variance explained by covariates	Metabolite variance explained by genetic variants	Metabolite variance explained by covariates
FEV_1_pp	3-Amino-2-piperidone	2	−0.005	0.006	rs37369	Missense variant	*AGXT2*	0.8	10.8	18.9	4
FEV_1_pp	(S)-a-Amino-omega-caprolactam	2	−0.005	0.006	NA	NA	*NA*	0.8	10.8	NA	NA
FEV_1_pp	Tetradecadienedioate (C14:2-DC)^*^	19	−0.006	0.001	rs11626972	Upstream gene variant	*ACOT2*	13.2	3	5.5	2
FEV_1_pp	Decadienedioic acid (C10:2-DC)^**^	19	−0.006	0.001	rs58231493	Upstream gene variant	*ACOT2*	13.2	3	18.6	3.2
FEV_1_/FVC	1-Methylnicotinamide	3	0.734	0.029	rs1495741	Intergenic variant	*NAT2*	0.2	2.8	5.8	7
FEV_1_/FVC	Nicotinamide	3	0.734	0.029	NA	NA	*NA*	0.2	2.8	NA	NA
FEV_1_/FVC	*N*1-methyl-2-pyridone-5-carboxamide	3	0.734	0.029	NA	NA	*NA*	0.2	2.8	NA	NA
FEV_1_/FVC	2-Aminobutyrate	4	1.031	0.002	rs10168931	Intron variant	*NAT8*	0.1	1.8	8.5	2.3
FEV_1_/FVC	Gamma-glutamyl-2-aminobutyrate	4	1.031	0.002	NA	NA	*NA*	0.1	1.8	NA	NA
FEV_1_/FVC	Aspartate	10	1.299	0	rs750062	Upstream gene variant	*ASPG*	0.3	17	6.6	6.5
FEV_1_/FVC	Glutamate	10	1.299	0	NA	NA	*NA*	0.3	17	NA	NA
FEV_1_/FVC	Linoleoyl-arachidonoyl-glycerol (18:2/20:4) [2]^*^	18	0.916	0.015	NA	NA	*NA*	3.8	4.6	NA	NA
FEV_1_/FVC	Oleoyl-arachidonoyl-glycerol (18:1/20:4) [2]^*^	18	0.916	0.015	rs174567	Intron variant	*FADS2*	3.8	4.6	5	3.1

ModuleID, module ID within phenotype; module beta, beta estimate of change in modules based on 1 unit increase in phenotype; adjusted score, score (*p*-value) after multiple testing correction; most significant independent SNP, SNP most significantly associated with metabolite. NA, no SNPs significantly associated; consequence, VEP annotation of variant; closest gene, closest gene to SNP as mapped in VEP; module first PC variance explained by genetic variants, adjusted *r*^2^ of linear regression model with the first PC of the module and independent mQTL SNPs. Module first PC variance explained by covariates, adjusted *r*^2^ of linear regression model with the first PC of the module and covariates (see Materials and Methods section). Metabolite variance explained by genetic variants, adjusted *r*^2^ of linear regression model with metabolite and independent mQTL SNPs. Metabolite variance explained by covariates, adjusted *r*^2^ of linear regression model with metabolite and covariates (see Materials and Methods section).

^*^ Indicates compounds that have not been officially confirmed based on a standard, but Metabolon is confident in its identity; ^**^indicates a compound for which a standard is not available, but Metabolon is confident in its identity or the information provided; (#) or [#] indicates a compound that is a structural isomer of another compound in the Metabolon spectral library.

PC, principal component.

## Discussion

While COPD is a disease of the lungs, we find a strong systemic metabolomic signature in the blood even after adjusting for common risk factors such as smoking. This is consistent with observations that COPD is associated with extrapulmonary diseases such as cardiovascular disease, osteoporosis, muscle wasting, and insulin resistance. Many of the metabolomic signatures we identified are similar to those found in these diseases (e.g., sphingolipids in cardiovascular and metabolic disorders^64^ or bone remodeling,^65^ acylcarnitines in osteoporosis,^66^ and diacylglycerols in insulin resistance^67^), suggesting common systemic pathways are important in COPD pathogenesis.

The lone metabolite associated with exacerbation frequency, of TMAP, further exemplifies the potential systemic effects of COPD. Although the reported association is novel in COPD, TMAP was recently identified as a biomarker of chronic kidney disease,^68^ a comorbidity of COPD,^69^ with a similar inverse association between TMAP abundances and disease severity. Moreover, while the biologic origin of TMAP has not yet been identified, it is suggested that myosin light-chain (MLC) protein degradation results in the release of TMAP.^68^ Disruption in MLC isoforms has been observed in COPD subjects with reduced activity and low oxygen supply, yet further work is needed to understand the pathophysiology of TMAP in exacerbations.

Systemic mitochondrial dysfunction, heightened in lungs with cigarette smoke-induced inflammatory-oxidative stress, has been implicated in the pathology of emphysema.^7,70^ We found further support of this as citrate was uniquely associated with the percent emphysema phenotype in regression analyses, demonstrating potential TCA cycle dysregulation. The increased power of phenotype-driven GGM network analysis positively associated three lactosylceramides with percent emphysema.

Abnormalities in glycosphingolipid metabolism have been noted to be associated with COPD phenotypes. For example, there is evidence for correlation between glycobiosyl ceramides and COPD exacerbations,^6^ and that glucosyl ceramide synthase, which governs the first step in the glycosphingolipid metabolism, is an important determinant of cell fate of lung endothelial cells.^71^ Moreover, lactosylceramide accumulation was recently identified as a common pathogenic mechanism that induces apoptotic-inflammatory responses and aberrant-autophagy leading to emphysema.^72^

Lactosylceramides can directly inhibit electron chain complexes, which enhance the production of reactive oxidation species in the mitochondria, potentially leading to lung inflammation and airway remodeling characteristic of emphysema.^72,73^ As the initial products in the formation of glycosphingolipids (e.g., lactosylceramides) are upregulated in insulin-resistant patients, increased lactosylceramide abundance may demonstrate comorbid mitochondrial dysregulation in COPD and metabolic disorders.

Several other metabolites previously associated with insulin resistance and other metabolic disorders were concordantly associated with COPD phenotypes. These included aromatic amino acids (phenylalanines) and BCAAs with both percent emphysema and airflow obstruction, as well as diacylglycerols, gamma-glutamyl amino acids, sphingomyelins, and lipids involved in the fatty acid and phospholipid metabolism, specifically with airflow obstruction. Abnormal amino acid and lipid metabolism may result from reduced dietary intake, oxidative stress, and increased strain on respiratory muscles with anoxia, leading to an active metabolic COPD state in COPD patients.^74,75^ These results confirm the findings of smaller studies that have shown strong associations between phospholipid-derived sphingolipids and COPD,^6,76^ and a recent two-cohort population study (KORA and ARIC) with 4347 controls and 393 COPD subjects that identified similar associations with BCAAs, aromatic amino acids, and glutamine/glutamate metabolites.^8^

However, our study differed from the KORA and ARIC study, in that we found more associations with FEV_1_/FVC than FEV_1_pp, indicating a metabolic signature of airflow obstruction. This may be because COPDGene primarily enrolled current and former smokers (>10 and >20 pack-years, respectively), oversampled for COPD cases, was older, and included only NHW subjects (ARIC had many AA subjects). Although we adjusted for these variables in our analyses, age and smoking have strong influences on metabolome, and thus the generalizability might be limited.

While lifestyle behaviors (e.g., smoking) are important risk factors for COPD, there is evidence that as much as 37% of the variability in lung function is genetic.^13^ To explore this, we first sought to identify the genetic effect on the metabolome, detecting significant SNP-metabolite associations in 109 (11%) of the metabolites tested (similar to the 119 of 529 [22%] metabolites previously reported by Shin et al).^54^ The strongest association was between missense SNP rsrs2147896 in *PYROXD2* and *N*6-methyllysine (*p*=3.97×10^−146^). *PYROXD2* has been associated with lysine metabolites in other mWAS, including *N*6-methyllysine, as well as trimethylamine in urine and dimethylamine in plasma.^77^ Of the 79 independent loci identified with recursive conditioning, 47 novel SNPs were found, including rs58231493, an upstream variant of *ACOT2* (coding for Acyl-CoA Thioesterase 2) associated with decadienedioic acid (C10:2-DC)**.

One of the most promising met-QTLs, as it was significant across replication cohorts, was in *UGT1A* region and associated with bilirubin pathway metabolites. In the Framingham Heart Study Offspring cohort, those with higher bilirubin due to a genetic polymorphism affecting the *UGT1A1* enzyme of bilirubin metabolism (the enzyme defect that leads to Gilbert's syndrome) had one-third the risk of cardiovascular events compared to wild-type carriers with normal bilirubin concentrations.^78^ Higher levels of serum bilirubin have been inversely associated with the risk of COPD severity, progression, and mortality,^79,80^ and more recently, fewer COPD exacerbations.^81^ In *in vitro* and animal studies, bilirubin prevents oxidation of lipids, which may protect the COPD lung by inhibiting lipid peroxidation.^80,82^ Variability in bilirubin concentration has been previously associated with SNPs in *UGT1A* region.^83,84^ In our analysis, we found both bilirubin and biliverdin nearing the conservative Bonferroni significance threshold for associations with airflow obstruction phenotypes (*p*=3.02×10^−3^ and 6.83×10^−4^ with FEV_1_/FVC, respectively). Integrating the genetic associations suggests that the observed associations between bilirubin/biliverdin and COPD may be mediated through genetics.

While there appears to be a strong genetic effect on the metabolome overall, there is less evidence for the genetic regulation of COPD-associated metabolites. Only 10 of the metabolites associated with COPD phenotypes through regression or phenotype-driven network analysis also had met-QTL SNP associations. Moreover, it was only within the module containing the fatty acids decadienedioic acid (C10:2-DC)** and tetradecadienedioate (14:2-DC)* that more variance was explained by an upstream variant of *ACOT2* than environmental variables.

The protein that *ACOT2* codes for, Acyl-CoA thioesterase-2, has been shown to facilitate mitochondrial fatty acid oxidation in mouse models and may warrant further study.^85^ The lack of evidence for genetic regulation in the COPD metabolome may implicate other downstream regulation (e.g., methylation, post-translational modification, and metabolism of exogenous metabolites) having a greater effect. Thus, modifiable behaviors, like smoking, diet, and exercise, may have a greater effect on the COPD metabolism than genetic predisposition.

While this study was strengthened by the large number of subjects in a well-categorized cohort, there were several limitations. First, this analysis was performed using blood samples, as opposed to bronchial lavage fluid, which may better represent COPD phenotypes.^33^ It is well documented that the blood metabolome, across multiple metabolic pathways, is strongly affected by demographic factors, including age and sex,^51,86^ which we replicated in our initial exploratory analyses. COPD pathology begins in the lungs and then manifests as systemic dysregulation across several biologic tissues. However, the observed effects on the metabolome may still not be as pronounced within blood as the effects of age and sex.

Second, although this is one of the largest mWAS studies reported, 957 subjects are still small compared to clinical GWAS studies. The cohort was also restricted to NHW subjects, which limits generalizability over the entire population. Moreover, as many distinct and independent met-QTL SNPs were identified for many metabolites, there may be multiple mechanisms along genetic and metabolic pathways that influence observed metabolite intensities.

Another major challenge in metabolomics is cross-platform replication. Although we had two independent cohort metabolomics platforms available for replication and we identified similar met-QTLs across cohorts, the named metabolome features for these met-QTL metabolites used three different annotation techniques (Metabolon was proprietary annotation; COPDGene—Emory used xMSannotator; and CU used Agilent MassHunter and IDBrowser). These annotation strategies are optimized to the platforms and cross-annotation was challenging.

Despite these challenges, we were able to use the presence of common met-QTLs as evidence to support a specific annotation; however, since all the platforms were untargeted, it was sometimes unclear which of the three annotations was the correct annotation. Finally, the sample sizes our COPDGene—Emory and replication cohorts, as well as their limited metabolite annotation, greatly limited our statistical power to detect replicating met-QTLs. Further work with targeted metabolomics studies could assist with these met-QTL associations.

In conclusion, this study found evidence in the blood metabolome for systemic dysregulation of metabolic pathways affecting COPD phenotypes in a diseased population. By further assessing the blood metabolome for genetic regulation, we reproduced several known associations and identified many novel met-QTL SNPs. Furthermore, we expanded and contextualized metabolite associations through COPD phenotype-driven module identification, integrating the genetic associations into the network view. While we found nongenetic factors to explain more variance in COPD-associated metabolites than genetic, further work is needed, potentially integrating the metabolome with other omics data types (e.g., epigenomics and proteomics), to elucidate and characterize dysregulated pathways in COPD pathogenesis.

## Supplementary Material

Supplemental data

Supplemental data

Supplemental data

Supplemental data

Supplemental data

Supplemental data

Supplemental data

## COPDGene investigators: core units

Administrative Center: James D. Crapo, MD (PI); Edwin K. Silverman, MD, PhD (PI); Barry J. Make, MD; and Elizabeth A. Regan, MD, PhD.

Genetic Analysis Center: Terri Beaty, PhD; Ferdouse Begum, PhD; Peter J. Castaldi, MD, MSc; Michael Cho, MD; Dawn L. DeMeo, MD, MPH; Adel R. Boueiz, MD; Marilyn G. Foreman, MD, MS; Eitan Halper-Stromberg; Lystra P. Hayden, MD, MMSc; Craig P. Hersh, MD, MPH; Jacqueline Hetmanski, MS, MPH; Brian D. Hobbs, MD; John E. Hokanson, MPH, PhD; Nan Laird, PhD; Christoph Lange, PhD; Sharon M. Lutz, PhD; Merry-Lynn McDonald, PhD; Margaret M. Parker, PhD; Dmitry Prokopenko, PhD; Dandi Qiao, PhD; Elizabeth A. Regan, MD, PhD; Phuwanat Sakornsakolpat, MD; Edwin K. Silverman, MD, PhD; Emily S. Wan, MD; and Sungho Won, PhD.

Imaging Center: Juan Pablo Centeno; Jean-Paul Charbonnier, PhD; Harvey O. Coxson, PhD; Craig J. Galban, PhD; MeiLan K. Han, MD, MS; Eric A. Hoffman, Stephen Humphries, PhD; Francine L. Jacobson, MD, MPH; Philip F. Judy, PhD; Ella A. Kazerooni, MD; Alex Kluiber; David A. Lynch, MB; Pietro Nardelli, PhD; John D. Newell, Jr., MD; Aleena Notary; Andrea Oh, MD; Elizabeth A. Regan, MD, PhD; James C. Ross, PhD; Raul San Jose Estepar, PhD; Joyce Schroeder, MD; Jered Sieren; Berend C. Stoel, PhD; Juerg Tschirren, PhD; Edwin Van Beek, MD, PhD; Bram van Ginneken, PhD; Eva van Rikxoort, PhD; Gonzalo Vegas Sanchez-Ferrero, PhD; Lucas Veitel; George R. Washko, MD; and Carla G. Wilson, MS.

PFT QA Center, Salt Lake City, UT: Robert Jensen, PhD.

Data Coordinating Center and Biostatistics, National Jewish Health, Denver, CO: Douglas Everett, PhD; Jim Crooks, PhD; Katherine Pratte, PhD; Matt Strand, PhD; and Carla G. Wilson, MS.

Epidemiology Core, University of Colorado Anschutz Medical Campus, Aurora, CO: John E. Hokanson, MPH, PhD; Gregory Kinney, MPH, PhD; Sharon M. Lutz, PhD; and Kendra A. Young, PhD.

Mortality Adjudication Core: Surya P. Bhatt, MD; Jessica Bon, MD; Alejandro A. Diaz, MD, MPH; MeiLan K. Han, MD, MS; Barry Make, MD; Susan Murray, ScD; Elizabeth Regan, MD; Xavier Soler, MD; and Carla G. Wilson, MS.

Biomarker Core: Russell P. Bowler, MD, PhD; Katerina Kechris, PhD; and Farnoush Banaei-Kashani, PhD.

## COPDGene investigators: clinical centers

Ann Arbor VA: Jeffrey L. Curtis, MD; Perry G. Pernicano, MD.

Baylor College of Medicine, Houston, TX: Nicola Hanania, MD, MS; Mustafa Atik, MD; Aladin Boriek, PhD; Kalpatha Guntupalli, MD; Elizabeth Guy, MD; and Amit Parulekar, MD.

Brigham and Women's Hospital, Boston, MA: Dawn L. DeMeo, MD, MPH; Alejandro A. Diaz, MD, MPH; Lystra P. Hayden, MD; Brian D. Hobbs, MD; Craig Hersh, MD, MPH; Francine L. Jacobson, MD, MPH; and George Washko, MD.

Columbia University, New York, NY: R. Graham Barr, MD, DrPH; John Austin, MD; Belinda D'Souza, MD; and Byron Thomashow, MD.

Duke University Medical Center, Durham, NC: Neil MacIntyre, Jr., MD; H. Page McAdams, MD; and Lacey Washington, MD.

HealthPartners Research Institute, Minneapolis, MN: Charlene McEvoy, MD, MPH, and Joseph Tashjian, MD.

Johns Hopkins University, Baltimore, MD: Robert Wise, MD; Robert Brown, MD; Nadia N. Hansel, MD, MPH; Karen Horton, MD; Allison Lambert, MD, MHS; and Nirupama Putcha, MD, MHS.

Lundquist Institute for Biomedical Innovationat Harbor UCLA Medical Center, Torrance, CA: Richard Casaburi, PhD, MD; Alessandra Adami, PhD; Matthew Budoff, MD; Hans Fischer, MD; Janos Porszasz, MD, PhD; Harry Rossiter, PhD; and William Stringer, MD.

Michael E. DeBakey VAMC, Houston, TX: Amir Sharafkhaneh, MD, PhD, and Charlie Lan, DO.

Minneapolis VA: Christine Wendt, MD; Brian Bell, MD; and Ken M. Kunisaki, MD, MS.

Morehouse School of Medicine, Atlanta, GA: Marilyn G. Foreman, MD, MS; Eugene Berkowitz, MD, PhD; and Gloria Westney, MD, MS.

National Jewish Health, Denver, CO: Russell Bowler, MD, PhD, and David A. Lynch, MB.

Reliant Medical Group, Worcester, MA: Richard Rosiello, MD, and David Pace, MD.

Temple University, Philadelphia, PA: Gerard Criner, MD; David Ciccolella, MD; Francis Cordova, MD; Chandra Dass, MD; Gilbert D'Alonzo, DO; Parag Desai, MD; Michael Jacobs, PharmD; Steven Kelsen, MD, PhD; Victor Kim, MD; A. James Mamary, MD; Nathaniel Marchetti, DO; Aditi Satti, MD; Kartik Shenoy, MD; Robert M. Steiner, MD; Alex Swift, MD; Irene Swift, MD; and Maria Elena Vega-Sanchez, MD.

University of Alabama, Birmingham, AL: Mark Dransfield, MD; William Bailey, MD; Surya P. Bhatt, MD; Anand Iyer, MD; Hrudaya Nath, MD; and J. Michael Wells, MD.

University of California, San Diego, CA: Douglas Conrad, MD; Xavier Soler, MD, PhD; and Andrew Yen, MD.

University of Iowa, Iowa City, IA: Alejandro P. Comellas, MD; Karin F. Hoth, PhD; John Newell, Jr., MD; and Brad Thompson, MD.

University of Michigan, Ann Arbor, MI: MeiLan K. Han, MD MS; Ella Kazerooni, MD MS; Wassim Labaki, MD MS; Craig Galban, PhD; and Dharshan Vummidi, MD.

University of Minnesota, Minneapolis, MN: Joanne Billings, MD; Abbie Begnaud, MD; and Tadashi Allen, MD.

University of Pittsburgh, Pittsburgh, PA: Frank Sciurba, MD; Jessica Bon, MD; Divay Chandra, MD, MSc; Carl Fuhrman, MD; and Joel Weissfeld, MD, MPH.

University of Texas Health, San Antonio, San Antonio, TX: Antonio Anzueto, MD; Sandra Adams, MD; Diego Maselli-Caceres, MD; Mario E. Ruiz, MD; and Harjinder Singh.

The authors thank the SPIROMICS participants and participating physicians, investigators, and staff for making this research possible. The authors thank the SPIROMICS participants and participating physicians, investigators, and staff for making this research possible. More information about the study and how to access SPIROMICS data is at www.spiromics.org. We would like to acknowledge the following current and former investigators of the SPIROMICS sites and reading centers: Neil E. Alexis, MD; Wayne H. Anderson, PhD; Mehrdad Arjomandi, MD; Igor Barjaktarevic, MD, PhD; R. Graham Barr, MD, DrPH; Lori A. Bateman, MSc; Surya P. Bhatt, MD; Eugene R. Bleecker, MD; Richard C. Boucher, MD; Russell P. Bowler, MD, PhD; Stephanie A. Christenson, MD; Alejandro P. Comellas, MD; Christopher B. Cooper, MD, PhD; David J. Couper, PhD; Gerard J. Criner, MD; Ronald G. Crystal, MD; Jeffrey L. Curtis, MD; Claire M. Doerschuk, MD; Mark T. Dransfield, MD; Brad Drummond, MD; Christine M. Freeman, PhD; Craig Galban, PhD; MeiLan K. Han, MD, MS; Nadia N. Hansel, MD, MPH; Annette T. Hastie, PhD; Eric A. Hoffman, PhD; Yvonne Huang, MD; Robert J. Kaner, MD; Richard E. Kanner, MD; Eric C. Kleerup, MD; Jerry A. Krishnan, MD, PhD; Lisa M. LaVange, PhD; Stephen C. Lazarus, MD; Fernando J. Martinez, MD, MS; Deborah A. Meyers, PhD; Wendy C. Moore, MD; John D. Newell Jr., MD; Robert Paine, III, MD; Laura Paulin, MD, MHS; Stephen P. Peters, MD, PhD; Cheryl Pirozzi, MD; Nirupama Putcha, MD, MHS; Elizabeth C. Oelsner, MD, MPH; Wanda K. O'Neal, PhD; Victor E. Ortega, MD, PhD; Sanjeev Raman, MBBS, MD; Stephen I. Rennard, MD; Donald P. Tashkin, MD; J. Michael Wells, MD; Robert A. Wise, MD; and Prescott G. Woodruff, MD, MPH. The project officers from the Lung Division of the National Heart, Lung, and Blood Institute were Lisa Postow, PhD, and Lisa Viviano, BSN; SPIROMICS was supported by contracts from the NIH/NHLBI (HHSN268200900013C, HHSN268200900014C, HHSN268200900015C, HHSN268200900016C, HHSN268200900017C, HHSN268200900018C, HHSN268200900019C, and HHSN268200900020C), grants from the NIH/NHLBI (U01 HL137880 and U24 HL141762), and supplemented by contributions made through the Foundation for the NIH and the COPD Foundation from AstraZeneca/MedImmune; Bayer; Bellerophon Therapeutics; Boehringer-Ingelheim Pharmaceuticals, Inc.; Chiesi Farmaceutici S.p.A.; Forest Research Institute, Inc.; GlaxoSmithKline; Grifols Therapeutics, Inc.; Ikaria, Inc.; Novartis Pharmaceuticals Corporation; Nycomed GmbH; ProterixBio; Regeneron Pharmaceuticals, Inc.; Sanofi; Sunovion; Takeda Pharmaceutical Company; and Theravance Biopharma and Mylan.

## Notes

**Cite this article as:** Gillenwater LA, Pratte KA, Hobbs BD, Cho MH, Zhuang Y, Halper-Stromberg E, Cruickshank-Quinn C, Reisdorph N, Petrache I, Labaki WW, O'Neal WK, Ortega VE, Jones DP, Uppal K, Jacobson S, Michelotti G, Wendt CH, Kechris KJ, Bowler RP (2020) Plasma metabolomic signatures of chronic obstructive pulmonary disease and the impact of genetic variants on phenotype-driven modules, *Network and Systems Medicine* 3:1, 159–181, DOI: 10.1089/nsm.2020.0009.

## Abbreviations Used

AA: African American
AE: anion exchange
AMRT: accurate mass and retention time
BCAA: branched chain amino acid
COPD: chronic obstructive pulmonary disease
CT: computed tomography
ESI: electrospray ionization
FAM: fatty acid metabolism
FEV_1_: forced expiratory volume at one second
FEV_1_pp: FEV_1_ percent predicted
FVC: forced vital capacity
GGM: Gaussian graphical model
GPI: glycophosphatidylinositol
GWAS: genome-wide association study
HILIC: hydrophilic interaction liquid chromatography
LC-MS/MS: liquid chromatography/tandem mass spectrometry
Met: metabolism
MLC: myosin light chain
mWAS: metabolome-wide association study
NHW: non-Hispanic white
PC: principal component
QTOF: quadrupole time-of-flight
RI: retention indices
RP/UPLC-MS/MS: reverse-phase/ultrahigh-performance liquid chromatography/tandem mass spectrometry
SNP: single-nucleotide polymorphism
TMAP: *N*,*N*,*N*-trimethyl-alanylproline betaine
UTR: untranslated region
VEP: Variant Effect Predictor
