# Supplementary material for: Plasma Metabolomic Signatures of Chronic Obstructive Pulmonary Disease and the Impact of Genetic Variants on Phenotype-Driven Modules
Source: Netw Syst Med. 2020 Dec 31;3(1):159–81. doi: 10.1089/nsm.2020.0009 (PMC8109053; doi:10.1089/nsm.2020.0009)
Supplement: Supplemental data [file Supp_FigS1.docx]

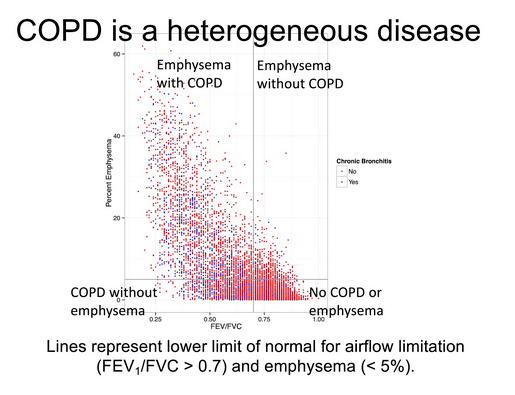


**Figure S1.** Scatter plot indicating the heterogeneity of COPD diagnosis. The x-axis represents FEV_1_/FVC and the y-axis indicates percent emphysema. The dot color indicates whether a subject has chronic bronchitis.
