# Supplementary material for: Plasma Metabolomic Signatures of Chronic Obstructive Pulmonary Disease and the Impact of Genetic Variants on Phenotype-Driven Modules
Source: Netw Syst Med. 2020 Dec 31;3(1):159–81. doi: 10.1089/nsm.2020.0009 (PMC8109053; doi:10.1089/nsm.2020.0009)
Supplement: Supplemental data [file Supp_FigS2.docx]

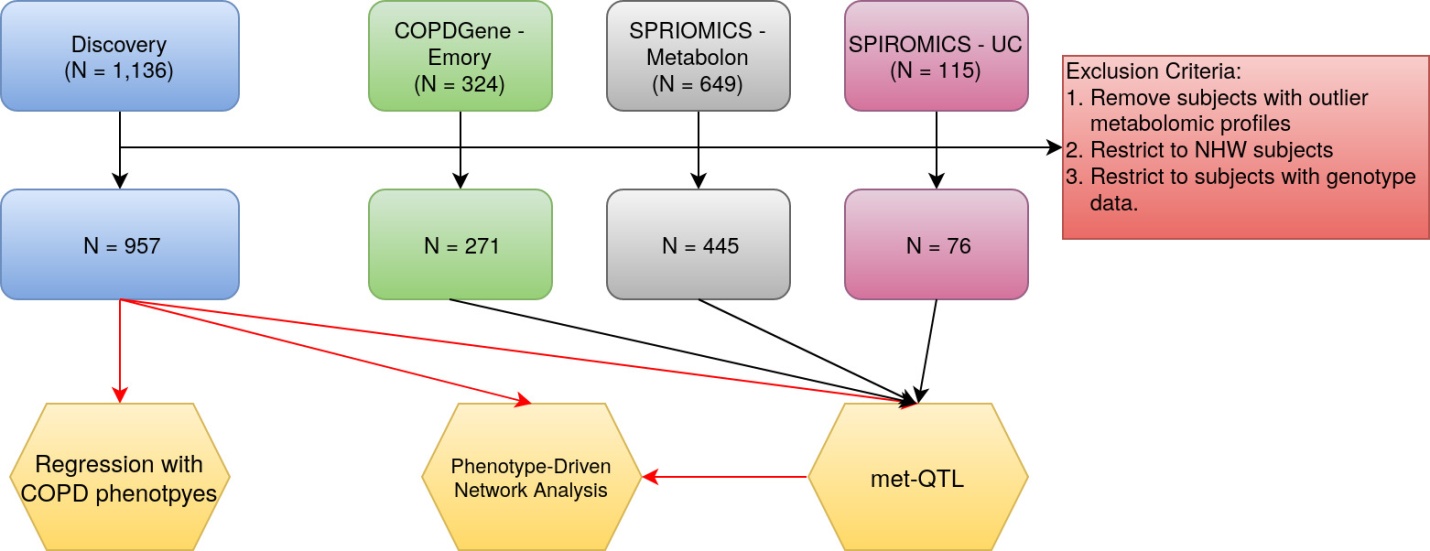
**Figure S2.** This study schematic outlines the cohorts involved in the analysis. The red arrows indicate the analyses performed with the Discovery cohort. Separate met-QTL analyses were performed on all populations to test for replication associated SNPs.
