# Supplementary material for: Plasma Metabolomic Signatures of Chronic Obstructive Pulmonary Disease and the Impact of Genetic Variants on Phenotype-Driven Modules
Source: Netw Syst Med. 2020 Dec 31;3(1):159–81. doi: 10.1089/nsm.2020.0009 (PMC8109053; doi:10.1089/nsm.2020.0009)
Supplement: Supplemental data [file Supp_FigS3.docx]

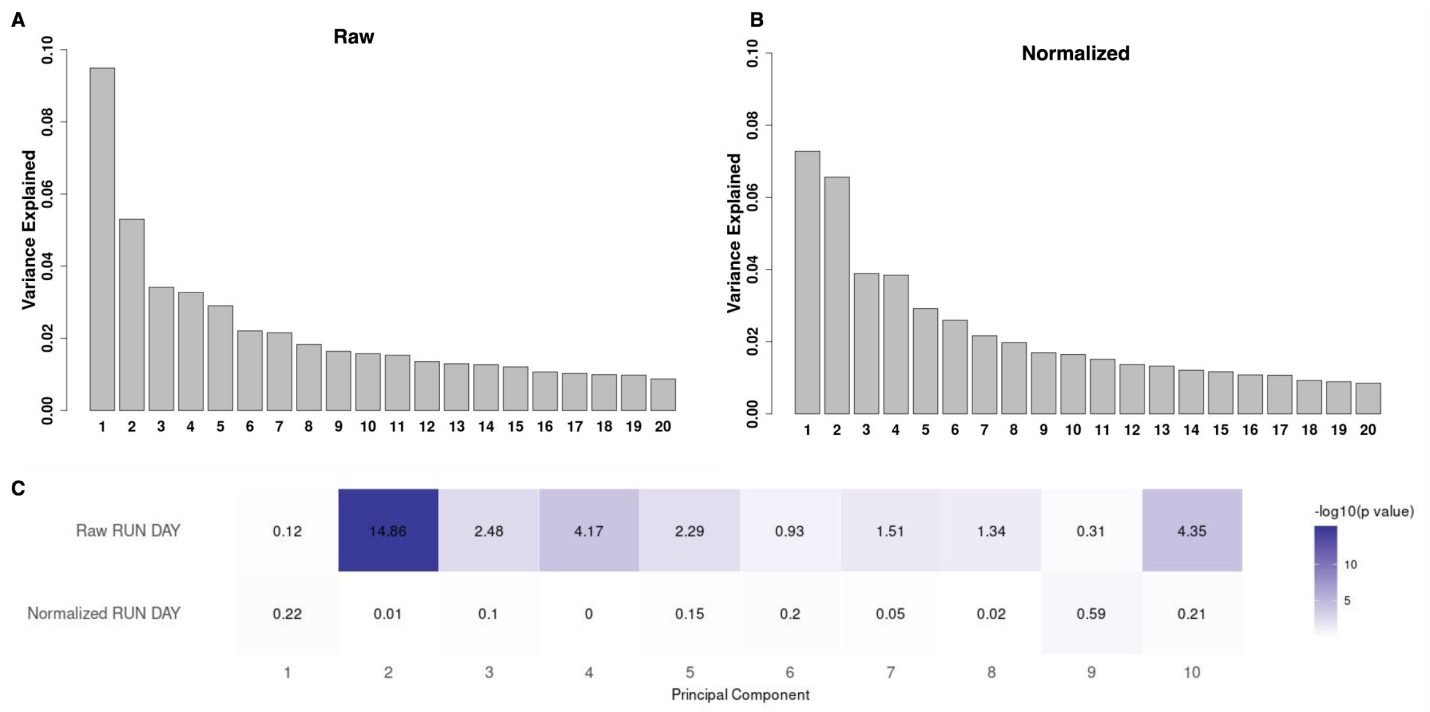


**Figure S3.** PCA of raw and normalized datasets. **A-B** Scree plots of the raw data and normalized data, respectively. The x axes represent the 20 PCs that explain the most variance, in decreasing order. The y axis is the proportion of variance explained. **C** Heatmap of the -log10(p values) from ANOVA with the principal component as the outcome and sample run day as independent variable.
