# Supplementary material for: Plasma Metabolomic Signatures of Chronic Obstructive Pulmonary Disease and the Impact of Genetic Variants on Phenotype-Driven Modules
Source: Netw Syst Med. 2020 Dec 31;3(1):159–81. doi: 10.1089/nsm.2020.0009 (PMC8109053; doi:10.1089/nsm.2020.0009)
Supplement: Supplemental data [file Supp_FigS4.docx]

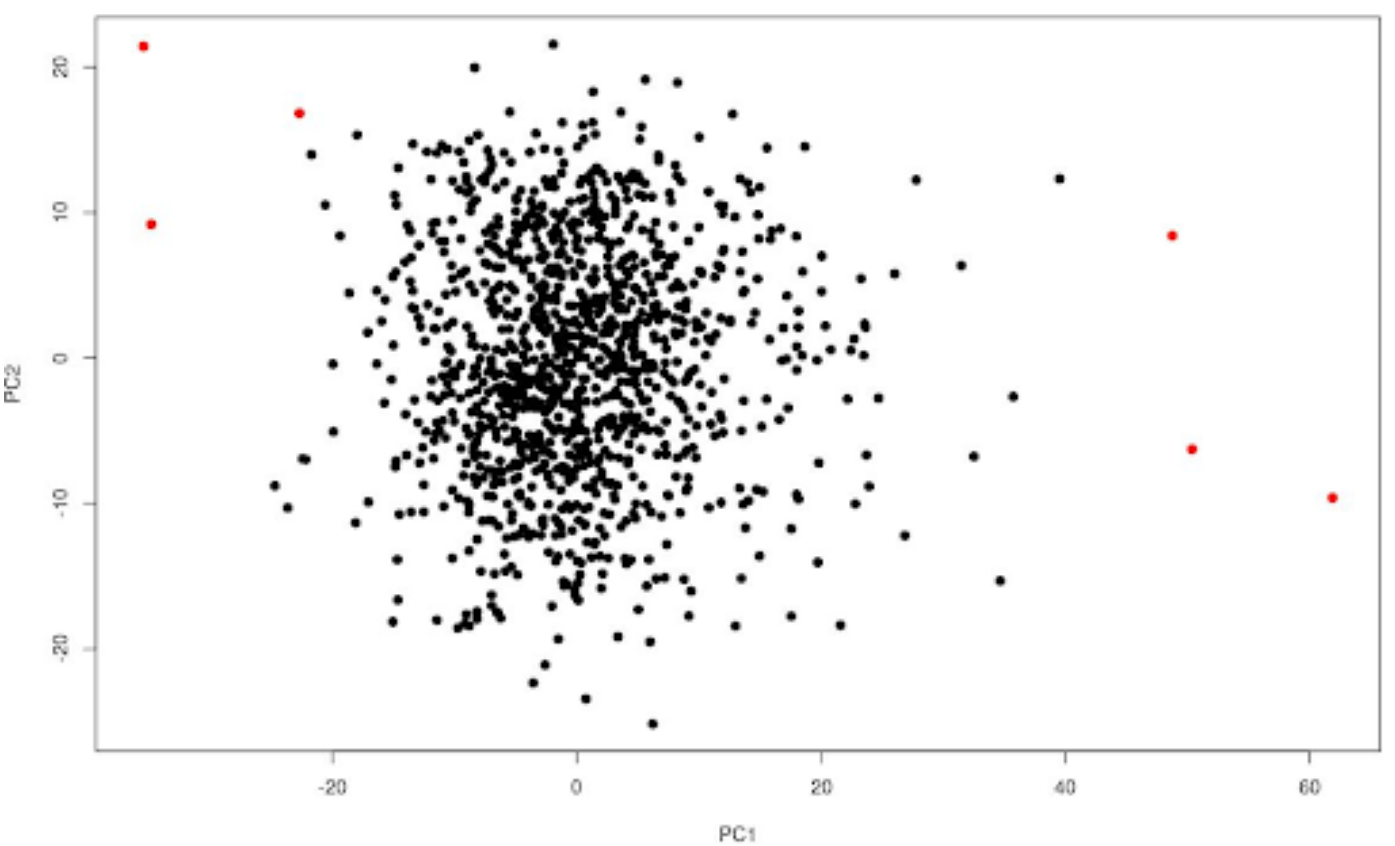


**Figure S4.** Removal of six subjects whose profiles were significantly different from other subjects. First two principal components with red circles indicating subjects removed. The highlighted red IQRs indicate the outlier subjects removed.
