# Supplementary material for: Plasma Metabolomic Signatures of Chronic Obstructive Pulmonary Disease and the Impact of Genetic Variants on Phenotype-Driven Modules
Source: Netw Syst Med. 2020 Dec 31;3(1):159–81. doi: 10.1089/nsm.2020.0009 (PMC8109053; doi:10.1089/nsm.2020.0009)
Supplement: Supplemental data [file Supp_FigS5.docx]

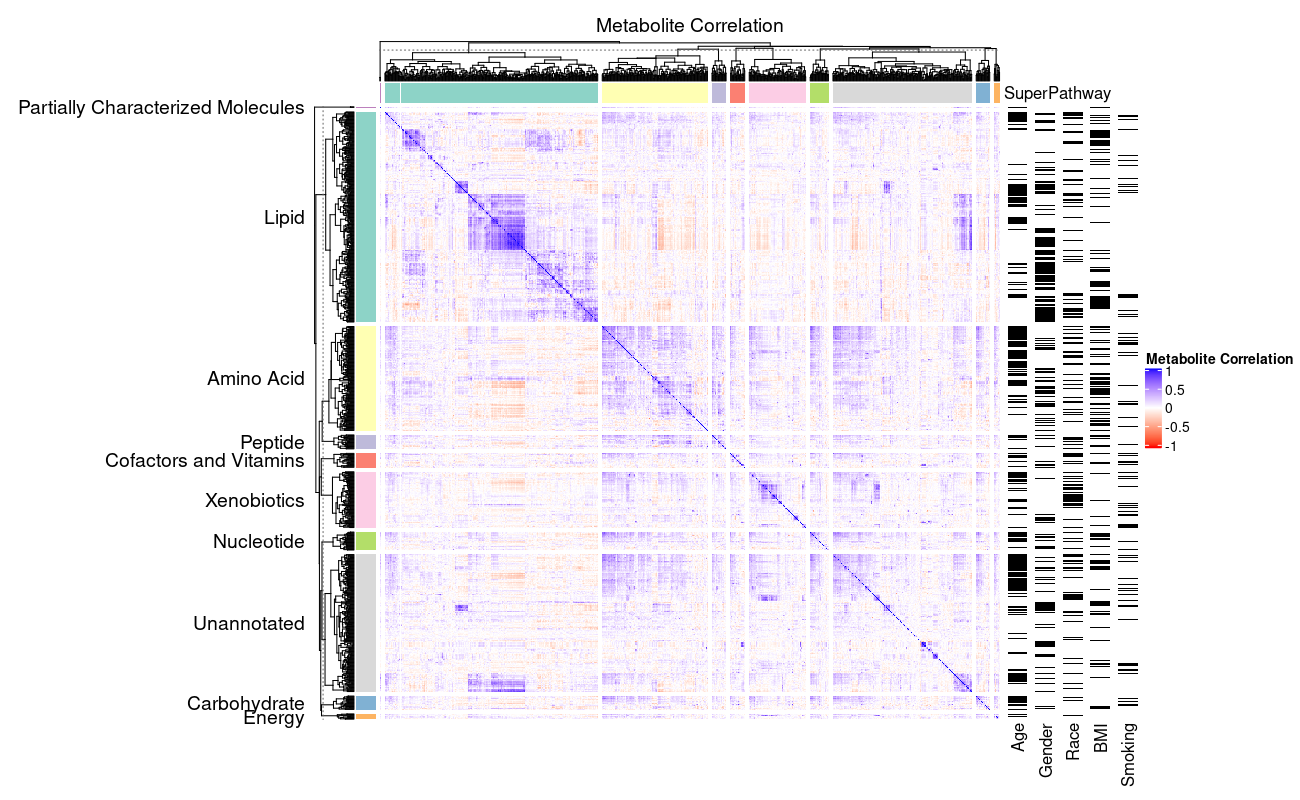


**Figure S5.** Hierarchical heat map showing pairwise correlations between metabolites. Metabolites are separated by and hierarchically clustered within the 10 super pathways (shown on left) annotated by Metabolon (Durham, USA). The black bands for age, sex (gender), race, BMI, and smoking indicate statistically significant p values, after accounting for false discovery rate from multiple testing, of individual metabolites with the respective clinical variables.
