# Supplementary material for: Plasma Metabolomic Signatures of Chronic Obstructive Pulmonary Disease and the Impact of Genetic Variants on Phenotype-Driven Modules
Source: Netw Syst Med. 2020 Dec 31;3(1):159–81. doi: 10.1089/nsm.2020.0009 (PMC8109053; doi:10.1089/nsm.2020.0009)
Supplement: Supplemental data [file Supp_FigS6.docx]

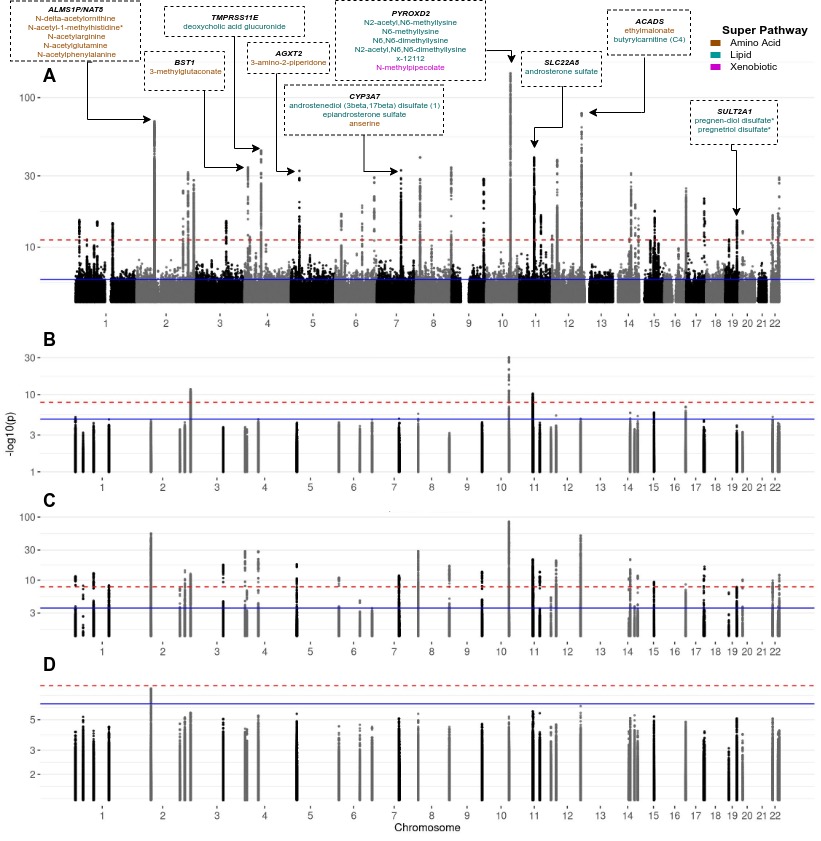


**Figure S6. Genome wide associations between single nucleotide polymorphisms (SNPs) and metabolites across replication cohorts.** Manhattan plot showing -log10 p-values from mWAS tests. The blue and red dashed lines indicate FDR and bonferroni significance, respectively. **A.** *Discovery* *mWAS* Loci in which >20% of the metabolite variance is explained by a single SNP are labelled by nearest gene and metabolites affected. Metabolite text colors coded by Super Pathway. **B.** *Validation mWAS.* **C.** *SPIROMICS – Metabolon mWAS. D****.*** *SPIROMICS – UC mWAS.*
